# FSH regulates fat accumulation and redistribution in aging through the Gαi/Ca^2+^/CREB pathway

**DOI:** 10.1111/acel.12331

**Published:** 2015-03-06

**Authors:** Xin-Mei Liu, Hsiao Chang Chan, Guo-Lian Ding, Jie Cai, Yang Song, Ting-Ting Wang, Dan Zhang, Hui Chen, Mei Kuen Yu, Yan-Ting Wu, Fan Qu, Ye Liu, Yong-Chao Lu, Eli Y Adashi, Jian-Zhong Sheng, He-Feng Huang

**Affiliations:** 1 International Peace Maternity and Child Health Hospital, School of Medicine, Shanghai Jiao Tong UniversityShanghai, China; 2 Department of Pathology & Pathophysiology, School of Medicine, Zhejiang UniversityZhejiang, China; 3 Shanghai Jiao Tong University – The Chinese University of Hong Kong Joint Research Center for Human Reproduction and Related DiseasesShanghai, China; 4 Epithelial Cell Biology Research Center, School of Biomedical Sciences, Faculty of Medicine, The Chinese University of Hong KongHong Kong, Hong Kong; 5 Key Laboratory for Regenerative Medicine (Jinan University – The Chinese University of Hong Kong), Ministry of EducationHangzhou, China; 6 The Key Laboratory of Reproductive Genetics, Ministry of EducationHangzhou, China; 7 Ningbo Maternal and Child Health HospitalZhejiang, China; 8 The Warren Alpert Medical School, Brown UniversityProvidence, RI, USA

**Keywords:** ageing, ca^2+^, endocrinology, mouse models, signal transduction, signalling

## Abstract

Increased fat mass and fat redistribution are commonly observed in aging populations worldwide. Although decreased circulating levels of sex hormones, androgens and oestrogens have been observed, the exact mechanism of fat accumulation and redistribution during aging remains obscure. In this study, the receptor of follicle-stimulating hormone (FSH), a gonadotropin that increases sharply and persistently with aging in both males and females, is functionally expressed in human and mouse fat tissues and adipocytes. Follicle-stimulating hormone was found to promote lipid biosynthesis and lipid droplet formation; FSH could also alter the secretion of leptin and adiponectin, but not hyperplasia, *in vitro* and *in vivo*. The effects of FSH are mediated by FSH receptors coupled to the Gαi protein; as a result, Ca^2+^ influx is stimulated, cAMP-response-element-binding protein is phosphorylated, and an array of genes involved in lipid biosynthesis is activated. The present findings depict the potential of FSH receptor-mediated lipodystrophy of adipose tissues in aging. Our results also reveal the mechanism of fat accumulation and redistribution during aging of males and females.

## Introduction

Major changes in fat mass and distribution occur during aging (Kuk *et al*., 2009). For instance, aging subjects contain higher amounts of body fat than young adults; furthermore, fat distributions of aging subjects are different from that of young adults, in which more visceral accumulation than subcutaneous depots is observed in the former than in the latter (Kuk *et al*., 2009). Likewise, ectopic fat accumulation occurs in bone marrow, muscle and liver and possibly contributes to age-related dysfunctions of these tissues (Rasouli *et al*., 2007). The accumulation of visceral (abdominal) adiposity in aging populations may be accompanied with insulin resistance, hypertension and dyslipidemia [hypertriglyceridemia, reduced high-density lipoprotein and low-density lipoprotein (LDL) particles] (Carr, 2003); in turn, risks of metabolic syndrome-related diseases, such as coronary heart disease, obesity, diabetes, cancer and cognitive dysfunction, in aging populations are increased.

Among many factors implicated in age-related fat accumulation and redistribution, the endocrine system possibly plays a major role (Rejnmark, 2013). For instance, androgens and oestrogens have been implicated in regulating ectopic fat deposition and regional fat distribution (Dieudonne *et al*., 1998; Pedersen *et al*., 2004; Finkelstein *et al*., 2013). Oestrogen activity has been considered a potential modulator of adiposity in males and females because oestrogen deficiency in males is possibly responsible for body fat increase, in addition to reduced sexual function (Finkelstein *et al*., 2013). Age-related changes in ectopic fat deposition and distribution have frequently been attributed to a decrease in circulating levels of androgens in males and oestrogens in females. However, the exact roles of sex hormones in regulating adipose tissues remain unclear.

Follicle-stimulating hormone, a glycoprotein hormone derived from the pituitary, is involved in reproductive events, such as gonadal and germ cell development, as well as sex hormone production (Simoni *et al*., 1997). As such, these functionalities are dependent on the binding of FSH to cognate FSH receptors (FSHRs) in target organs, such as the ovary and the testis. Circulating FSH levels also undergo dramatic changes with age because of the loss of negative feedback from inhibin, as well as androgens and oestrogens, in aging males and females, respectively (Ausmanas *et al*., 2007). For example, high circulating FSH levels are among the diagnostic signs of menopause in females. Circulating FSH levels similarly show an age-related increase in males (Tajar *et al*., 2012). FSHR expression was first thought to be restricted to the gonads. However, further studies have detected FSHR in osteoclasts, suggesting a possible role of FSH in postmenopausal osteoporosis (Sun *et al*., 2006). Interestingly, FSHR has been found in the adipose tissue of chickens; hence, FSHR has been implicated in lipid biosynthesis (Cui *et al*., 2012).

The possible role of FSH and FSHR in fat accumulation and redistribution in humans during aging has not been investigated. Considering the observed correlation between high circulating FSH levels and increased body weight of Chinese males and females prompted us to hypothesize that FSHR might be expressed in human adipocytes (fat cells) and that high circulating FSH levels might modulate adipocyte functions during aging. Therefore, we performed this study to test this hypothesis.

## Results

### Age-dependent changes in circulating FSH levels and their correlation with body mass index (BMI) of Chinese males and females

In the clinical investigation of females (*n = *9853), circulating FSH levels of postmenopausal females are found to be approximately 10-fold higher than those of premenopausal counterparts. In the clinical investigation of males (*n = *8736), circulating FSH levels in males > 60 years were more than threefold higher than those of males aged < 45 years (Tables1 and 2). In further investigation involving 413 males (aged 61–65 years) and 499 females (aged 51–55 years), circulating FSH levels were correlated with the increase in BMI (ΔBMI) of males (Fig.1A, *r* = 0.553, *P *<* *0.0001) and females (Fig.1B, *r* = 0.710, *P *<* *0.0001).

**Table 1:** Serum FSH levels in females of different age groups

Groups	Child-bearing age (*n *=* *4779)	Postmenopause (*n *=* *5074)
FSH (IU L^−1^)	6.50 ± 0.04	59.52 ± 0.30**
Age (year)	30.04 ± 0.07	56.73 ± 0.08**

Data are represented as mean ± SEM.

** *P* < 0.001.

**Table 2:** Serum FSH levels in males of different age groups

Age (years)	20–45 (*n = *7681)	46–59 (*n = *567)	60–70 (*n = *488)
FSH (IU L^−1^)	4.82 ± 0.30	8.79 ± 0.28**	15.50 ± 0.54**^,^##

Data are represented as mean ± SEM.

** *P* < 0.001 compared with males aged 20–45 years;

## *P* < 0.001 compared with males aged 46–59 years.

**Fig 1:**
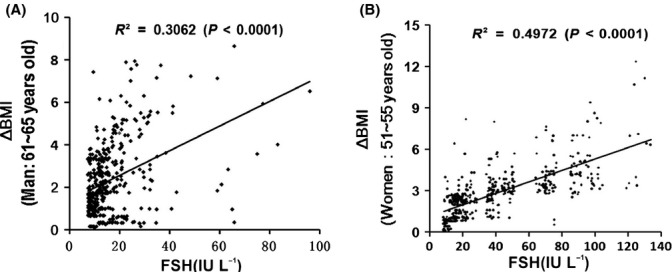
Correlation between FSH levels and change in BMI (ΔBMI) in aging males and females. (A) Correlation between FSH levels and increase in BMI (ΔBMI) in 414 males (aged 61–65 years). Pearson's *r *=* *0.553, *P *<* *0.0001. (B) Correlation between FSH levels and increase in BMI (ΔBMI) in 499 females (51–55 years). Pearson's *r = *0.710, *P *<* *0.0001. ΔBMI_(males)_ = BMI_(present)_ − BMI_(aged 35–45 years)_; ΔBMI_(females)_ = BMI_(post-menopausal)_ − BMI_(pre-menopausal)_.

### FSHR expression in human and mouse adipose tissues and cells

**Fig 2:**
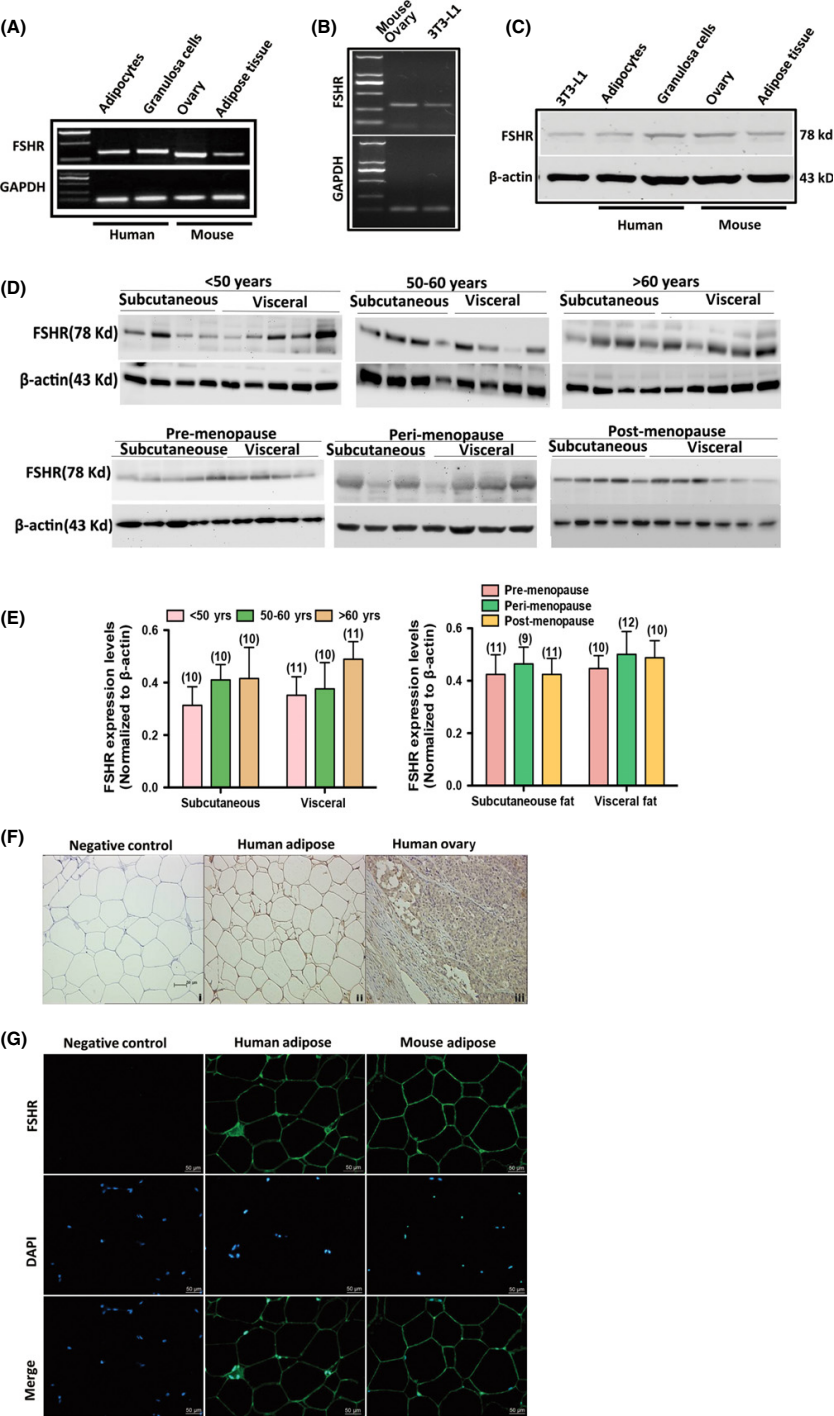
Expression and localization of FSHR in human and mouse adipocytes. (A) mRNA expressions of FSHR in adipocytes and granulosa cells of human and in adipose tissue and ovarian tissue of mouse. GAPDH served as loading control. (B) mRNA expression of FSHR in 3T3-L1 preadipocytes. GAPDH served as loading control. (C) Protein expressions of FSHR in 3T3-L1 preadipocytes, adipocytes and granulosa cells of human and in adipose tissues and ovarian tissues of mouse. β-actin served as loading control. (D) Protein expression of FSHR in subcutaneous and visceral fat of males aged < 50, 50–60 and > 60 years and in pre-, peri- and postmenopausal females. (E) Relative protein levels of FSHR in subcutaneous and visceral fat of pre-, peri- and postmenopausal females. Values are mean ± SEM; the number of samples in each group is shown at the bottom of the column. No significant differences were observed between groups. (F) Localization of FSHR in human adipose and ovarian tissues by immunohistochemistry. (G) Localization of FSHR in human and mouse adipose tissues by immunofluorescence. Nuclei were stained with DAPI.

Conducting RT–PCR and Western blot, we found the expected bands of FSHR in human and mouse adipose tissues (Fig.2A,C); we also found the expected bands of FSHR in 3T3-L1 mouse preadipocyte cell line (Fig.2B,C). By sequencing, we confirmed that the PCR product was FSHR. The target nucleotide sequences were submitted to GenBank with the accession number JN003607 (Fig. S1). We further observed that FSHR was expressed in subcutaneous and visceral fat. No significant differences in FSHR expression levels were detected among males aged < 50, 50–60 and > 60 years in either subcutaneous fat (*P *=* *0.6439) or visceral fat (*P *=* *0.424; Fig.2D,E). Likewise, no significant differences in FSHR expression levels were observed among pre-, peri- and postmenopausal females in either subcutaneous fat (*P *=* *0.8969) or visceral fat (*P *=* *0.859). By immunohistochemical and immunofluorescent staining, we observed that FSHR was localized in the cell membrane of adipocytes (Fig.2F,G).

### Follicle-stimulating hormone promoted fat storage and lipogenesis *in vitro*

**Fig 3:**
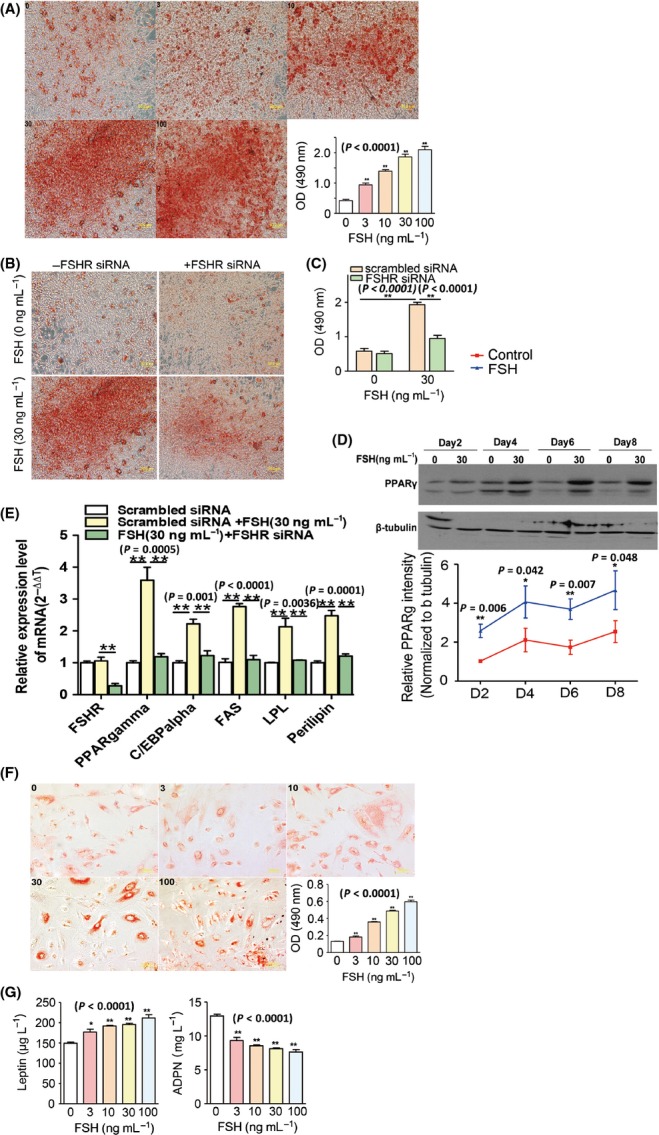
FSH-enhanced lipid biosynthesis in 3T3-L1 and human preadipocytes. (A) Concentration-dependent effects of FSH (0–100 ng mL^−1^) on the differentiation of 3T3-L1 preadipocytes at 8 days. (B, C) Effects of FSHR-targeted siRNA treatment on the differentiation of FSH-induced 3T3-L1 preadipocytes at 8 days. (D) PPARγ expression in FSH-induced 3T3-L1 preadipocytes at 2–8 days of differentiation. (E) PPARγ, C/EBPα, fatty acid synthase, lipoprotein lipase and perilipin transcript levels in FSH-induced 3T3-L1 preadipocytes with or without FSHR-specific siRNA treatment. (F) Concentration-dependent effect of FSH on the differentiation of human preadipocytes at 8 days. (G) Leptin and adiponectin levels in the culture medium of FSH-induced human preadipocyte differentiation at 8 days. Values are mean ± SEM (*n *=* *5 for A–G). *P <* *0.05 and **P <* *0.01.

Follicle-stimulating hormone was administered to insulin-IBMX-hexadecadrol-primed 3T3-L1 preadipocytes; this treatment accelerated the formation of lipid droplets in a concentration-dependent manner (Fig.3A, *P* *<* 0.0001). This process was then disrupted by the knockdown of FSHR with a specific siRNA (Fig.3B,C; Fig. S2). Furthermore, the upregulation of an array of critical genes involved in lipid biosynthesis was induced in FSH-treated 3T3-L1 preadipocytes; in particular, the genes involved in the synthesis of fatty acids (FAs) and triglycerides (TGs), including peroxisome proliferator-activated receptor gamma (PPARγ; Fig.3D,E), CAAT/enhanced binding proteins (C/EBPα), fatty acid synthase (FAS), lipoprotein lipase (LPL) and perilipin (Fig.3E), were upregulated. Nevertheless, this process could be reversed by the knockdown of FSHR-targeted siRNA (Fig.3E). Likewise, FSH was administered to primary human adipocytes which exhibited an accelerated lipid droplet formation (Fig.3F, *P* *<* 0.0001). Follicle-stimulating hormone also induced concentration-dependent changes in primary human adipocytes by secretion of increased leptin levels (*P < *0.0001) and decreased adiponectin (ADPN) levels (*P < *0.0001; Fig.3G). However, flow cytometry results showed that FSH treatment did not alter cell cycle phase distribution of 3T3-L1 preadipocytes; no significant biphasic change in the rate of cell cycle progression was observed between FSH-treated preadipocytes and control cells (Fig. S3).

### Effects of high FSH levels on fat accumulation and distribution in mouse models

**Fig 4:**
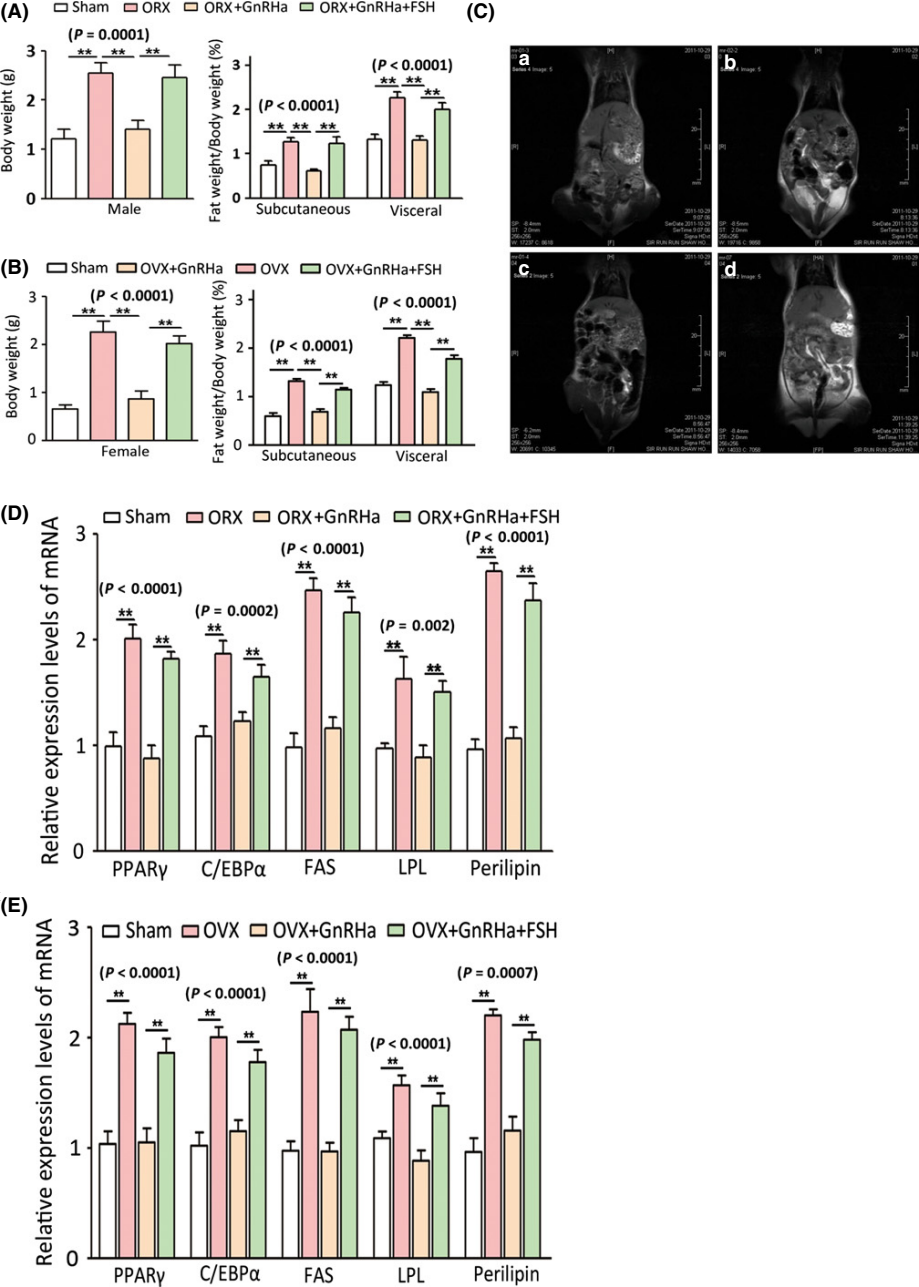
Induction of obesity phenotypes and regulation of pro-adipogenic genes by high FSH levels in mice. (A) Body weight variance and subcutaneous and visceral fat mass of male mice in different treatment groups (ORX, orchiectomy). (B) Body weight variance and subcutaneous and visceral fat mass of male mice in different treatment groups (OVX, ovariectomy). (C) Coronal T1-weighted spin-echo MR images obtained with volume segmentation of intra-abdominal tissues from total adipose tissue (a, sham group; b, OVX group; c, OVX+GnRHa group; and d, OVX+GnRHa+FSH group). (D) Transcript levels of lipid biosynthesis-related genes in adipocytes of male mice. (E) Transcript levels of lipid biosynthesis-related genes in adipocytes of female mice. Values are mean ± SEM (*n *=* *10 for A and B, *n *=* *5 for C–F). *P *<* *0.05 and **P <* *0.01.

We further explored whether high circulating FSH levels affect fat accumulation and distribution in mice. To mimic high circulating FSH levels during aging, we subjected male and female mice to surgical gonadectomy (Hsueh & Erickson, 1979). After surgery was conducted, radioimmunoassay was performed to confirm the increase in serum FSH and LH and the decrease in serum testosterone and oestrogen (Fig. S4A–D). Concurrently, significant increases in body weight and fat mass were noted (Fig.4A,B). Gonadectomy-induced gains in body weight and fat mass were suppressed by treatment with a gonadotropin-releasing hormone (GnRH) agonist (GnRHa; 0.5 μg day^−1^ for 4 weeks; Fig.4A,B); high FSH and LH levels (Fig. S4A,B) were also suppressed by persistent low testosterone and oestrogen levels (Fig. S4C,D). To evaluate the possible effect of LH on body weight and fat mass, we treated gonadectomized mice with recombinant FSH (0.15 IU day^−1^) and GnRHa. Follicle-stimulating hormone treatment in the absence of LH resulted in a significant increase in body weight and fat mass (Fig.4A,B). The effect of FSH on fat accumulation was further confirmed by magnetic resonance imaging of the adipose volume of gonadectomized mice. The fat mass of OVX/ORX mice (with higher FSH and LH levels) and OVX/ORX+GnRHa+FSH-treated mice (higher FSH but lower LH levels than sham control mice) was significantly increased (Figs4C and S5).

Male and female subcutaneous fats, including those found in arm, flank, thigh and abdominal fat storage, and visceral fats, including perirenal, epicardial, retroperitoneal, omental and mesenteric fat depots, showed a significant increase in adipocyte cell size after the mice were subjected to gonadectomy; this increase was reversed by GnRHa treatment (Fig. S6A,B). A similar increase in adipocyte cell size was also observed in gonadectomized FSH+GnRHa-treated mice. In male and female mice, adipocyte cell size was altered to a greater extent in visceral fat than in subcutaneous fat (Fig. S6A,B). This observation is consistent with the characteristic of greater fat accumulation in visceral depots than in subcutaneous fat depots during aging. Gonadectomy/GnRHa/FSH treatment also altered the serum levels of a series of molecules related to fat metabolism and functions; among these molecules were TG, total cholesterol (Tch), leptin and ADPN (Fig. S7). In particular, a number of genes known to play key roles in promoting lipid biosynthesis (FAs and TG synthesis) from preadipocytes to adipocytes, including PPARγ, C/EBPα, FAS, LPL and perilipin, were significantly increased in adipose tissues after gonadectomy was performed. These gonadectomy-induced increments were abolished by GnRHa but mimicked by FSH with GnRHa administration (Fig.4D,E).

### FSHR is coupled to the Gαi subunit triggering Ca^2+^-dependent signalling pathway

**Fig 5:**
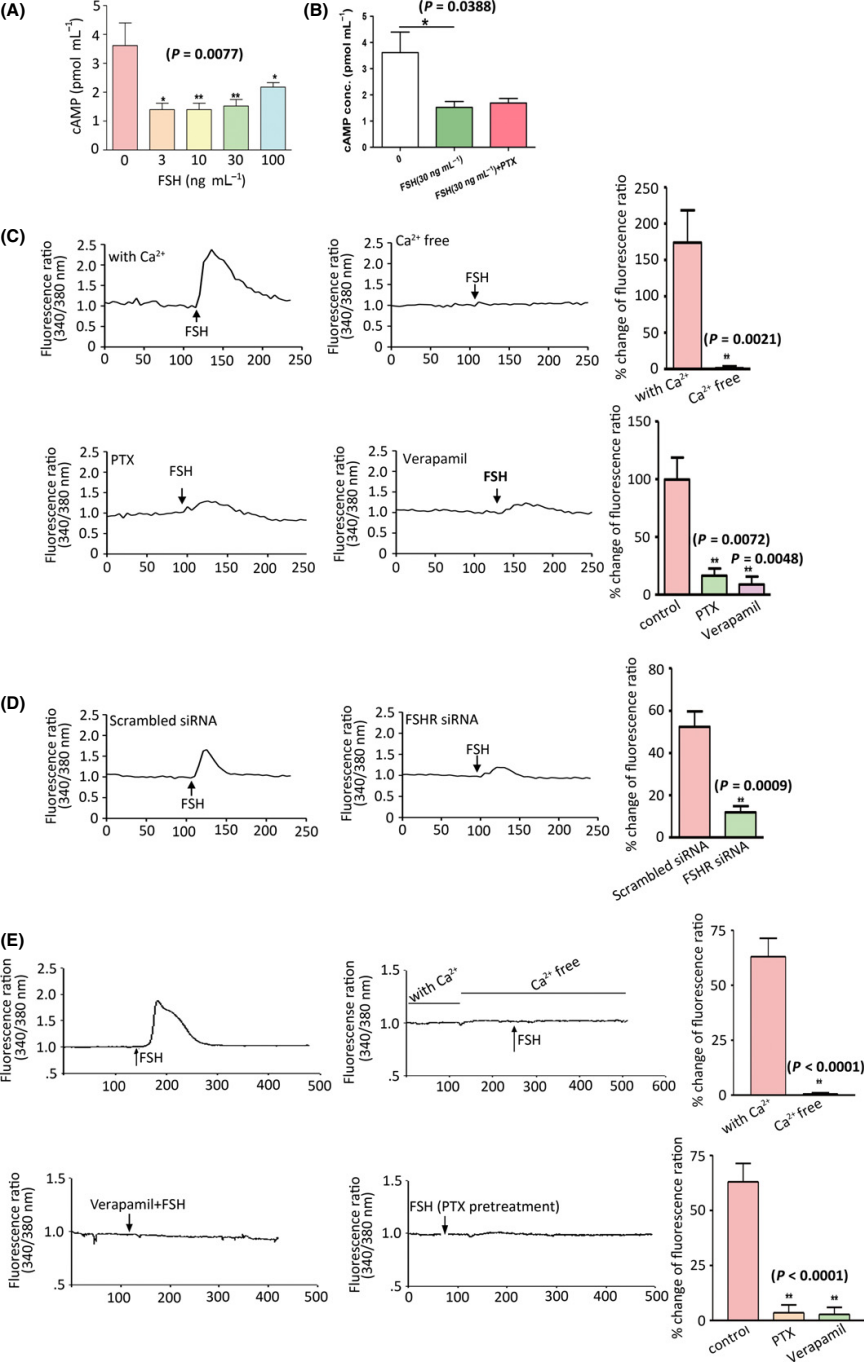
Coupling of FSH receptor to Gαi protein and Ca^2+^ influx in preadipocytes. (A) Intracellular cAMP levels in FSH-induced 3T3-L1 preadipocytes. (B) Intracellular cAMP levels in FSH (30 ng mL^−1^)-induced 3T3-L1 cells with and without pertussis toxin (PTX). (C) Intracellular Ca^2+^ measurements in FSH (300 ng mL^−1^)-treated 3T3-L1 preadipocytes with and without Ca^2+^ and with PTX (100 nm) and verapamil (20 μm) treatment. (D) Intracellular Ca^2+^ measurements in FSH (300 ng mL^−1^)-treated 3T3-L1 preadipocytes with scrambled siRNA and specific FSHR siRNA (7 nm) treatment. (E) Intracellular Ca^2+^ measurements in FSH-induced human adipocytes with and without Ca^2+^ and PTX (100 nm) or verapamil (20 μm) treatment. Values are mean ± SEM (*n *=* *5 for A–E). *P <* *0.05, **P <* *0.01.

To explore FSHR-mediated signalling pathway, we initially investigated whether cyclic AMP (cAMP) is involved in FSH-induced lipid biosynthesis in 3T3-L1 preadipocytes because gonadal FSHR is coupled to cAMP activation (Conti, 2002). However, FSH treatment did not activate cAMP accumulation in 3T3-L1 preadipocytes; instead, FSH treatment suppressed cAMP accumulation (*P = *0.0116) even with or without pertussis toxin (PTX, 100 nm), a Gαi inhibitor (Fig.5A,B). We then explored whether Ca^2+^ is involved in this process. Follicle-stimulating hormone treatment (30 ng mL^−1^) triggered an increase in intracellular Ca^2+^ concentrations in 3T3-L1 preadipocytes (Fig.5D). Moreover, this effect was all but abolished when extracellular Ca^2+^ was removed and substantially inhibited when verapamil (20 μm), a Ca^2+^ channel blocker, was applied; this result suggested that FSH activated an extracellular Ca^2+^-dependent signal transduction mechanism in FSH-promoted lipid synthesis. Considering that the FSHR-coupled Gαi subunit is known to trigger Ca^2+^-dependent signalling, we also investigated the effect of PTX (100 nm) on FSH-induced Ca^2+^ increase in 3T3-L1 cells. The results showed that PTX significantly reduced the effect of FSH (Fig.5D). Furthermore, FSH-induced Ca^2+^ increase was significantly reduced by the knockdown of FSHR (Fig.5E). Similar experiments performed on human primary fat cells yield similar results (Fig.5F). Thus, we obtained human primary fat cells and found that FSH (30 ng mL^−1^)-induced increase in intracellular Ca^2+^ concentrations was abolished when extracellular Ca^2+^, verapamil (20 μm) and PTX (100 nm) were removed.

**Fig 6:**
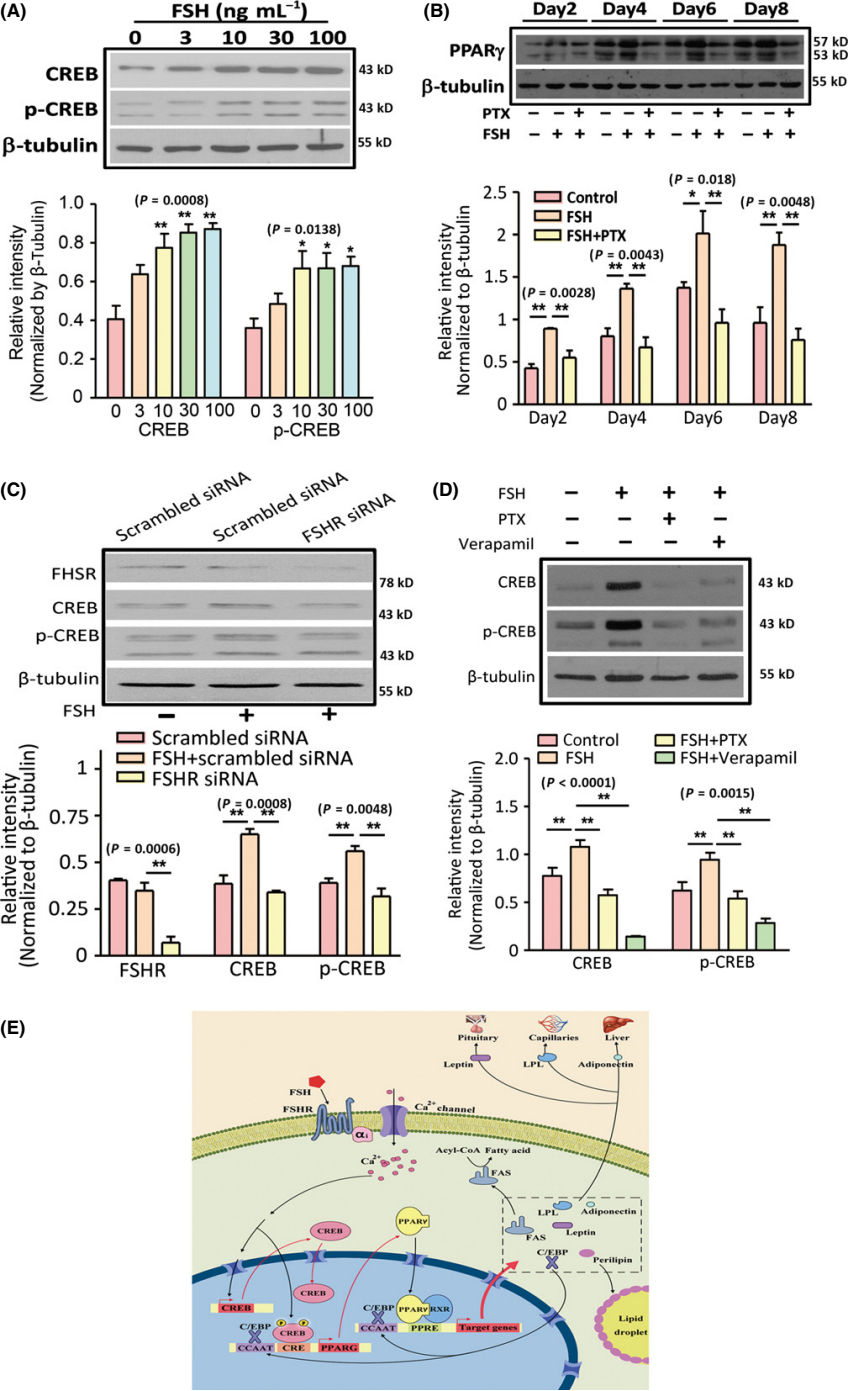
Follicle-stimulating hormone-induced pro-lipogenic gene expression via Gαi/Ca^2+^-dependent CREB/PPARγ pathway in 3T3-L1 preadipocytes. (A) Total CREB and p-CREB expressions in 3T3-L1 cells treated with FSH (0–100 ng mL^−1^) for 30 min. (B) PPARγ expression in FSH-induced 3T3-L1 cells at 2–8 days of differentiation with or without pertussis toxin (PTX) (100 nm) treatment. (C) FSHR, CREB and p-CREB expressions in 3T3-L1 cells treated with FSH for 30 min with or without FSHR-specific siRNA treatment. (D) CREB and p-CREB expression in 3T3-L1 cells treated with FSH for 30 min with PTX (100 nm) or verapamil (20 μm). (E) Working model of FSH-induced lipid biosynthesis in adipocytes. Values are mean ± SEM (*n *=* *5 for A–D). *P <* *0.05 and **P <* *0.01.

We then determined whether FSH-induced Ca^2+^-dependent signalling is indeed involved in FSH-promoted lipid biosynthesis. Phosphorylated cAMP-response-element-binding protein (p-CREB) and total CREB expression, as well as PPARγ expression, were stimulated by FSH (Fig.6A,B); these effects were inhibited by the knockdown of FSHR-targeted siRNA (Fig.6C) or PTX and verapamil (Fig.6D). This result indicated that FSHR mediated FSH-induced pro-synthesis of lipids via the Ca^2+^–CREB pathway; thus, the genes involved in FA and TG synthesis were upregulated, and lipid biosynthesis in adipocytes was promoted (Fig.6E).

## Discussion

Although studies have shown that FSH levels are drastically increased during aging (Feldman *et al*., 2002; Ausmanas *et al*., 2007), especially in postmenopausal females, studies on the contributions of FSH to aging processes are scarce. For instance, Chu *et al*. (2003) investigated the relationship between basal FSH level and fasting lipoprotein profiles of premenopausal females with normal menstrual cycles. Chu *et al*. (2003) found that premenopausal females with an FSH level ≥ 7 IU L^−1^ exhibit significantly increased TC and LDL compared with those with FSH < 7 IU L^−1^; this result suggests that increased FSH levels are correlated with known cardiovascular disease (CVD) risk factors and reveals the critical roles of FSH in lipid metabolism. Our present study demonstrates that FSH levels are positively correlated with changes in BMI (ΔBMI) of males and females; this result also suggests the possible role of high FSH levels in fat mass increase and redistribution in aging subjects.

Follicle-stimulating hormone binds to FSHRs localized in cell membranes. FSHRs are glycoprotein hormone receptors (GpHRs), a subset of the G protein-coupled receptor (GPCR) family. In this study, FSHR in human adipocytes was characterized as homologous with FSHRs in other cell types; however, FSHR in human adipocytes differed from FSHRs in other cell types with regard to Asn to Ser at position 680. In other cell types, this mutation was previously reported as a single nucleotide polymorphism (Cargill *et al*., 1999). In another previous study on Sertoli cells in 288 healthy males, FSHR polymorphism of Asn-Ser at position 680 exhibits a distribution frequency of 19% (Asatiani *et al*., 2002). FSHR in mature adipocytes displays typical features of GpHR family members characterized by a large extracellular domain with multiple, imperfect, leucine-rich repeats activated by specific ligands (Hsu *et al*., 2000).

Using a preadipocyte 3T3-L1 model *in vitro* (Chakrabarti *et al*., 2010), we demonstrated that FSH directly stimulated the formation of lipid droplets in adipocytes, but not adipocyte hyperplasia. Adipose tissue is well equipped to handle and store excess lipids. However, the expandability of adipose tissue is a limited process (Virtue & Vidal-Puig, 2010). Synthesized lipids likely exceed the expanded volume of adipose tissues, but adipose tissues do not have the necessary storage space (Finkelstein *et al*., 2013); as a consequence, toxic lipid spillover into nonadipose cells occurs, thereby inducing a decreased clearance of TG-rich lipoprotein particles and stimulating fat deposition in nonadipose tissue (Chavez & Summers, 2010; Lafontan, 2014). Therefore, the major cause of aging-related ectopic fat deposition is the reduced ability of adipose tissues to store lipids (Cartwright *et al*., 2007). Aging is also associated with increased lipid spillover into nonadipose tissues, including muscle, liver and bone; this increased lipid spillover potentially contributes to dysfunction of these organs (Kirkland *et al*., 2002). Although the mechanism of aging-related reduced ability to store lipids in adipose tissues remains unclear, the expansion of adipose tissue hypertrophy induced by excessive lipid synthesis might be an underlying cause. Our results further demonstrated FSH-promoted lipid biosynthesis in adipocytes. In particular, FSH induced the upregulation of PPARγ, C/EBP, FAS, LPL and perilipin in adipocytes. Among these molecules, PPARγ and C/EBP might target the genes directly implicated in lipogenic pathways and lipid storage in adipose tissues, including LPL and FAS (Evans *et al*., 2004). LPL, synthesized in adipose tissue, is the gatekeeper of fat storage; when upregulated, LPL acts on lipoproteins and may generate increased FAs that are either transported into tissues or mixed with circulating FFAs via a spillover mechanism (Lafontan, 2014). As a key enzyme, FAS is involved in the successful biosynthesis of FAs and TGs; increased FAS not only contributes to FA generation but also affects FA oxidation (Jensen-Urstad & Semenkovich, 2012). In addition, perilipins in adipose tissue are involved in lipid droplet formation; increased lipids are associated with obesity (Wang *et al*., 2003; Kern *et al*., 2004; Brasaemle, 2007). Our results revealed that FSH induced the upregulation of PPAR, C/EBP, LPL, FAS and perilipin in adipocytes; this result suggested that FSH-induced excessive lipid biosynthesis in adipose tissue might cause lipodystrophy and cause spillover from adipocytes.

Follicle-stimulating hormone is involved in the lipodystrophy of adipose tissue; this involvement is further supported by the ability of FSH to alter the levels of two key indicators of lipid droplet formation, namely leptin and ADPN. Leptin is an indicator of total fat mass; thus, abnormal leptin levels are implicated in the pathophysiology of hypertension, atherosclerosis and coronary heart disease (Trujillo & Scherer, 2006; Lago *et al*., 2009; Sattar *et al*., 2009). ADPN is another adipokine derived from adipocytes; ADPN decreases during adiposity and increases after weight is reduced (Yang *et al*., 2001; Asayama *et al*., 2003). Decreased ADPN levels are possibly associated with intra-abdominal fat increase (Asayama *et al*., 2003; Cnop *et al*., 2003). Our findings showed that FSH induced dose-dependent leptin production and ADPN reduction *in vitro*; these results further suggested the potential role of FSH in fat accumulation and distribution in aging.

The expression levels of FSHR in adipocytes do not seem to vary significantly with age in males and females. This result suggests that changes in FSH levels in the blood, rather than changes in FSHR expression, are responsible for the regulation of lipid biosynthesis. Thus, increased FSH levels in aging population likely cause age-related fat accumulation and distribution. This notion is supported by the results obtained from gonadectomized mice, which provide an experimental model of high FSH levels observed in aging populations (Hsueh & Erickson, 1979). Despite persistent low testosterone and oestrogen levels, GnRHa reversed gonadectomy-induced changes in body weight and fat mass in mice. Hence, high FSH and/or LH levels are possibly responsible for fat accumulation and redistribution in aging populations (Ferrara *et al*., 2002; Moretti *et al*., 2005). However, the replenishment of recombinant FSH in GnRHa-treated gonadectomized mice resulted in a significant increase in body weight and fat mass, which excludes the possible effect of LH; this result affirmed that FSH is responsible for weight gain and fat accumulation. Similar to the results obtained *in vitro*, the results from gonadectomized mice also showed that FSH is responsible for the increased serum levels of TG, Tch, leptin and ADPN; furthermore, FSH is implicated in the increased expression of pro-lipogenic genes (PPARγ, C/EBP, FAS, LPL and perilipin) *in vivo*. These results indicated the contribution of FSH to fat accumulation in mice. Furthermore, FSH elicited a stronger effect on the cell size of visceral fat than on the cell size of subcutaneous fat; this result possibly indicated the shift from subcutaneous fat to visceral fat accumulation commonly observed in aging populations (Cartwright *et al*., 2007). Nevertheless, further studies should be conducted to confirm this possibility.

The effect of FSH on fat accumulation is likely mediated by the corresponding functional FSHR in adipocytes because the respective effect can be abolished or significantly reduced by the knockdown of FSHR. FSHR is expressed and implicated exclusively in gonads (Asatiani *et al*., 2002) by coupling to the Gαs subunit and activating the cAMP/PKA pathway.

However, we found that FSH in preadipocytes stimulated Ca^2+^ influx, which can be blocked by PTX; this result suggested that FSHR couples with PTX-sensitive (Gαi/o) G protein in adipocytes (Clancy *et al*., 2005). Interestingly, FSHR is expressed in osteoclasts and involved in postmenopausal osteoporosis via FSHR activation coupled to the Gαi subunit; this result provided the first evidence of FSHR expression and physiological function in organs other than the gonads in an age-related process (Sun *et al*., 2006). Therefore, FSHR may be involved in a broad range of aging processes in organs other than the gonads via a signalling mechanism distinct from that in the gonads.

This study demonstrated that FSH could promote lipid biosynthesis by the coupling of FSHR to Ca^2+^-dependent signalling pathway; as a result, CREB, a transcription factor known to elicit pleiotropic effects on lipid biosynthesis and fat accumulation, becomes activated and TG accumulates. Inactivated CREB causes adipocyte apoptosis (Reusch & Klemm, 2002). By contrast, activated CREB initiates transcriptional remodelling that activates a large number of adipose-related genes; in this process, PPARγ plays a major role in complex transcriptional cascades. For instance, PPARγ activation promotes terminal differentiation of preadipocytes by the induction of several genes, including C/EBP, FAS, LPL and perilipin, which are important for TG uptake and storage. *In vitro*, high FSH levels possibly resulted in an increase in lipid biosynthesis in adipocytes by upregulating CREB, triggering PPARγ and recruiting other key enzymes to promote fat accumulation during aging.

In conclusion, our findings revealed a potential role of FSH in promoting fat accumulation and redistribution in humans. Our results also provided a new molecular mechanism of age-related obesity. High levels of FSH, rather than decreased levels of sex hormones, are possibly responsible for fat accumulation and redistribution in aging populations of males and females. The stronger effect of FSH on the cell size of visceral fat than on the cell size of subcutaneous fat may explain the shift from subcutaneous fat to visceral fat accumulation commonly associated with aging (Zamboni *et al*., 2005). This shift reveals important implications because visceral obesity is a major risk factor of many age-related diseases (Huffman & Barzilai, 2009). Interestingly, FSHR was also expressed in other tissues, including skeletal muscle and liver (Fig. S8), where fat also accumulates during aging. Therefore, FSH may also be involved in age-related fat accumulation in these tissues. This study demonstrated that FSH was involved in lipid metabolism and related signalling pathway. Along with high FSH levels commonly observed in aging populations, FSHR may be considered a potential therapeutic target to reduce the risk of age-related obesity and diseases.

## Experimental procedures

### Subjects and study design

Animal holding and experimental procedures were performed in accordance with the institutional guidelines for laboratory animals established by the Animal Care and Use Committee, School of Medicine, Zhejiang University. We obtained informed consent from populations who participated in these studies at the affiliated hospitals of School of Medicine, Zhejiang University, with the approval of the institutional review board.

We collected basal FSH levels of 9853 females and 8736 males undergoing routine physical examinations at the affiliated hospitals of Zhejiang University from 1 January 2001 to 1 July 2011. We recruited 414 males aged 61–65 years and 499 females aged 51–55 years who participated in regular physical examination without (i) history of radiotherapy or chemotherapy, (ii) application of hormones, (iii) previous or current infertility treatment, (iv) endocrine disease, (v) operation of reproductive system and (vi) pregnancy. The correlation of present FSH levels and change in BMI [ΔBMI, ΔBMI = BMI (present) − BMI (previous)] of the human subjects was analysed. For aged females, ΔBMI = postmenopausal BMI − premenopausal BMI; for aged man, ΔBMI = BMI at 61–65 years − BMI at 35–45 years.

### Chemicals and reagents

Recombinant human FSH (Gonal-F) was purchased from Serono (Frankfurter Str. 25064293 Darmstadt, Germany). GnRHa (triptorelin acetate for injection) was purchased from Beaufour-Ipsen Pharmaceutical Co., Ltd. (Xiqing District, Tianjin, China). Hormone radioimmunoassay kits, including luteinizing hormone (LH, B04TFB), FSH (B03PZA) and testosterone (T, B11TBB), were purchased from Beijing North Institute of Biological Technology (Panjiamiao, Fengtan District, Beijing, China). Estradiol radioimmunoassay kit (E_2_, RG6) was purchased from Tianjin Nine Tripods Medical & Bioengineering Co., Ltd. (China). Antibodies used for immunoblotting included FSHR (Abcam, Cambridge, UK), PPARγ, CREB, phospho-CREB (Cell Signaling Technology, Beverly, MA, USA), β-actin and β-tubulin (Santa Cruz Biotechnology, Santa Cruz, CA, USA). All of the other chemicals were purchased from Sigma Chemical Co. (St. Louis, MO, USA).

### Radioimmunoassay

Blood samples were collected to estimate serum testosterone, estradiol, LH and FSH by radioimmunoassay. Serum samples were separated by standard procedures and stored at −20 °C for subsequent analysis. Testosterone, estradiol, LH and FSH were subjected to radioimmunoassay by double-antibody precipitation methods (Crowe *et al*., 1997). Measurements were carried out in duplicate. Samples and standard (100 μL) were preincubated overnight at 4 °C temperature with first antibody (100 μL). Tracer (100 μL; iodinated testosterone, estradiol, LH and FSH solutions; 12 000–15 000 cpm) was added to each tube, and the tubes were incubated for another 24 h at 4 °C. Secondary antibody (500 μL) was added and incubated for 0.5 h; water (500 μL) was then added. Bound and free tracers were separated by centrifugation (1600 *g*) for 5 min. The supernatant containing unbound tracer was aspirated using a vacuum pump; the bound tracer in the precipitate was counted using a gamma counter.

### Mouse preadipocyte culture, treatment and oil red O staining

3T3-L1 preadipocyte cell line was obtained from American Type Culture Collection (Manassas, VA, USA). For preadipocyte differentiation, confluent cultures of human preadipocytes and 3T3-L1 were exposed to an induction medium containing DMEM supplemented with dexamethasone (1 mm), insulin (5 mg mL^−1^), isobutylmethylxanthine (0.5 mm) and 10% FBS for 48 h. The cells were maintained in DMEM containing insulin (5 mg mL^−1^) and 10% FBS for 48 h and subsequently cultured in DMEM containing 10% FBS for several days (Zuk *et al*., 2002). Different FSH concentrations (Gonal-F; Serono) were added to the cells in the induction medium.

For oil red O staining, differentiated cells were washed twice with PBS and fixed with 10% formalin at 4 °C overnight. The fixed cells were maintained at room temperature for 2 h, washed twice with distilled water and stained with filtered oil red O solution (0.5% oil red O in isopropyl alcohol). The stained cells were observed under a microscope.

### Isolation and culture of human preadipocytes and adipocytes

Adipose tissues were obtained from patients undergoing abdominal surgery. Patients with diabetes, hypertension, endocrine diseases and malignant tumour were excluded. The samples were sliced into pieces with a diameter of 1 mm. The sliced tissue was then digested with 0.1% collagenase I (Gibco, Gaithersburg, MD, USA) in HBSS at 37 °C with intermittent shaking for 1.5 h. The cells were separated from undigested tissue mass using a sterilized 250-μm nylon mesh. Afterwards, the filtrate was centrifuged for 10 min at 800 *g* at room temperature. The cell pellet was washed twice. The erythrocytes were then lysed with 155 mm NH_4_Cl for 5 min. The cells were resuspended in DMEM supplemented with 10% FBS, plated at a density of 10^4^ cells cm^−2^ and incubated at 37 °C with 5% CO_2_. After 48 h, the medium was replaced to remove nonadherent cells.

### Animal experiments

Eight-week-old C57BL/6 mice were used in this study (Zhejiang University Animal Center, Hangzhou, China). The mice were housed in a 12-h:12-h light:dark cycle at 25 ± 0.5 °C and 50–60% humidity and fed *ad libitum* with standard diet (containing 10% fat) and water. The mice were weighed before the experiments were conducted. The 8-week-old female and male mice with body weight of 18–20 g were divided into four groups (*n *=* *10 per group): (i) sham operated, (ii) OVX/ORX, (iii) OVX/ORX+GnRHa and (iv) OVX/ORX+GnRHa+FSH. In the OVX/ORX group, the mice were gonadectomized to mimic high serum FSH levels in aging populations. In OVX/ORX+GnRHa groups, gonadectomized mice were treated with GnRHa (0.5 μg day^−1^, triptorelin acetate; Beaufour-Ipsen) by intraperitoneal injection for 4 weeks to inhibit pituitary gonadotropin secretion. In OVX/ORX+GnRHa+FSH groups, castrated mice were initially injected with GnRHa (0.5 μg day^−1^) for 2 weeks and then with human recombinant FSH (0.15 IU day^−1^; Gonal-F; Serono) and with GnRHa (0.5 μg day^−1^) for another 2 weeks.

Four weeks after surgery, the mice were re-weighed, and subcutaneous and visceral fat mass were measured. The mice were imaged using a BioSpec (Bruker Corporation, Beijing Office:5109 Everbright International Trust Mansion, 11 Zhongguancun South Street, Beijing,China) 3-T MRI system with a custom-sized send–receive coil. Coronal spin-echo T1-weighted sequences were obtained from the entire mouse (head to anus). The parameters of the coronal T1-weighted spin-echo images were listed as follows: TR/TE, 500/8; matrix size, 256 × 160; slice thickness, 2 mm; and number of excitations, 2 (Du *et al*., 2005).

### Statistics

Data were analysed using spss 16.0 for Windows, SPSS China, Xishu Software (Shanghai) Co.,Ltd., Yanandong Road, Huangpu District, Shanghai, China). In all of the histograms, error bars represent the standard error of the mean (SEM). Statistical comparisons were performed using Student's *t*-tests between two groups; one-way ANOVA with Tukey's multiple comparison test was conducted to analyse more than two experimental groups. Statistical significance was set at *P *<* *0.05. Pearson's correlation coefficient was determined to analyse the correlation between basal FSH levels and BMI of 913 aging individuals.

## Supporting Information

**Fig. S1** Molecular cloning of human FSHR gene coding sequence (CDS) in human adipocytes.

**Fig. S2** Relative expression of FSHR mRNA in 3T3-L1 pre-adipocytes treated by specific FSHR siRNA (7 nm).

**Fig. S3** Effects of FSH on 3T3-L1 cell cycle progression.

**Fig. S4** Serum levels of FSH, LH, testosterone and estogen after gonadectomy.

**Fig. S5** Coronal T1-weighted spin-echo MR images obtained with volume segmentation for intra-abdominal for total adipose tissue.

**Fig. S6** H–E staining of subcutaneous and visceral adipose tissue in different treatment groups with corresponding measurement of cell size in female (A) and male (B) mice.

**Fig. S7** Lipid metabolism of mice after gonadectomy.

**Fig. S8** Expression of FSHR mRNA in difference tissues.

**Table S1** primers in molecular cloning of human FSHR coding sequence. **Data S1** Supporting experimental procedures.

## References

[b1] Asatiani K, Gromoll J, Eckardstein SV, Zitzmann M, Nieschlag E, Simoni M (2002). Distribution and function of FSH receptor genetic variants in normal men. Andrologia.

[b2] Asayama K, Hayashibe H, Dobashi K, Uchida N, Nakane T, Kodera K, Shirahata A, Taniyama M (2003). Decrease in serum adiponectin level due to obesity and visceral fat accumulation in children. Obes. Res.

[b3] Ausmanas MK, Tan DA, Jaisamrarn U, Tian XW, Holinka CF (2007). Estradiol, FSH and LH profiles in nine ethnic groups of postmenopausal Asian females: the Pan-Asia Menopause (PAM) study. Climacteric.

[b4] Brasaemle DL (2007). Thematic review series: adipocyte biology. The perilipin family of structural lipid droplet proteins: stabilization of lipid droplets and control of lipolysis. J. Lipid Res.

[b5] Cargill M, Altshuler D, Ireland J, Sklar P, Ardlie K, Patil N, Shaw N, Lane CR, Lim EP, Kalyanaraman N, Nemesh J, Ziaugra L, Friedland L, Rolfe A, Warrington J, Lipshutz R, Daley GQ, Lander ES (1999). Characterization of single-nucleotide polymorphisms in coding regions of human genes. Nat. Genet.

[b6] Carr MC (2003). The emergence of the metabolic syndrome with menopause. J. Clin. Endocrinol. Metab.

[b7] Cartwright MJ, Tchkonia T, Kirkland JL (2007). Ageing in adipocytes: potential impact of inherent, depot-specific mechanisms. Exp. Gerontol.

[b8] Chakrabarti P, English T, Shi J, Smas CM, Kandror KV (2010). Mammalian target of rapamycin complex 1 suppresses lipolysis, stimulates lipogenesis, and promotes fat storage. Diabetes.

[b9] Chavez JA, Summers SA (2010). Lipid oversupply, selective insulin resistance, and lipotoxicity: molecular mechanisms. Biochim. Biophys. Acta.

[b10] Chu MC, Rath KM, Huie J, Taylor HS (2003). Elevated basal FSH in normal cycling women is associated with unfavourable lipid levels and increased cardiovascular risk. Hum. Reprod.

[b11] Clancy SM, Fowler CE, Finley M, Suen KF, Arrabit C, Berton F, Kosaza T, Casey PJ, Slesinger PA (2005). Pertussis-toxin-sensitive Galpha subunits selectively bind to C-terminal domain of neuronal GIRK channels: evidence for a heterotrimeric G-protein-channel complex. Mol. Cell Neurosci.

[b12] Cnop M, Havel PJ, Utzschneider KM, Carr DB, Sinha MK, Boyko EJ, Retzlaff BM, Knopp RH, Brunzell JD, Kahn SE (2003). Relationship of adiponectin to body fat distribution, insulin sensitivity and plasma lipoproteins: evidence for independent roles of age and sex. Diabetologia.

[b13] Conti M (2002). Specificity of the cyclic adenosine 3′,5′-monophosphate signal in granulosa cell function. Biol. Reprod.

[b14] Crowe MA, Padmanabhan V, Hynes N, Sunderland SJ, Enright WJ, Beitins IZ, Roche JF (1997). Validation of a sensitive radioimmunoassay to measure serum follicle-stimulating hormone in cattle: correlation with biological activity. Anim. Reprod. Sci.

[b15] Cui H, Zhao G, Liu R, Zheng M, Chen J, Wen J (2012). FSH stimulates lipid biosynthesis in chicken adipose tissue by upregulating the expression of its receptor FSHR. J. Lipid Res.

[b16] Dieudonne MN, Pecquery R, Boumediene A, Leneveu MC, Giudicelli Y (1998). Androgen receptors in human preadipocytes and adipocytes: regional specificities and regulation by sex steroids. Am. J. Physiol.

[b17] Du H, Dardzinski BJ, O'Brien KJ, Donnelly LF (2005). MRI of fat distribution in a mouse model of lysosomal acid lipase deficiency. AJR Am. J. Roentgenol.

[b18] Evans RM, Barish GD, Wang YX (2004). PPARs and the complex journey to obesity. Nat. Med.

[b19] Feldman HA, Longcope C, Derby CA, Johannes CB, Araujo AB, Coviello AD, Bremner WJ, McKinlay JB (2002). Age trends in the level of serum testosterone and other hormones in middle-aged men: longitudinal results from the Massachusetts male aging study. J. Clin. Endocrinol. Metab.

[b20] Ferrara CM, Lynch NA, Nicklas BJ, Ryan AS, Berman DM (2002). Differences in adipose tissue metabolism between postmenopausal and perimenopausal women. J. Clin. Endocrinol. Metab.

[b21] Finkelstein JS, Lee H, Burnett-Bowie SA, Pallais JC, Yu EW, Borges LF, Jones BF, Barry CV, Wulczyn KE, Thomas BJ, Leder BZ (2013). Gonadal steroids and body composition, strength, and sexual function in men. N. Engl. J. Med.

[b22] Hsu SY, Kudo M, Chen T, Nakabayashi K, Bhalla A, van der Spek PJ, van Duin M, Hsueh AJ (2000). The three subfamilies of leucine-rich repeat-containing G protein-coupled receptors (LGR): identification of LGR6 and LGR7 and the signaling mechanism for LGR7. Mol. Endocrinol.

[b23] Hsueh AJ, Erickson GF (1979). Extrapituitary action of gonadotropin-releasing hormone: direct inhibition ovarian steroidogenesis. Science.

[b24] Huffman DM, Barzilai N (2009). Role of visceral adipose tissue in aging. Biochim. Biophys. Acta.

[b25] Jensen-Urstad AP, Semenkovich CF (2012). Fatty acid synthase and liver triglyceride metabolism: housekeeper or messenger?. Biochim. Biophys. Acta.

[b26] Kern PA, Di Gregorio G, Lu T, Rassouli N, Ranganathan G (2004). Perilipin expression in human adipose tissue is elevated with obesity. J. Clin. Endocrinol. Metab.

[b27] Kirkland JL, Tchkonia T, Pirtskhalava T, Han J, Karagiannides I (2002). Adipogenesis and aging: does aging make fat go MAD?. Exp. Gerontol.

[b28] Kuk JL, Saunders TJ, Davidson LE, Ross R (2009). Age-related changes in total and regional fat distribution. Ageing Res. Rev.

[b29] Lafontan M (2014). Adipose tissue and adipocyte dysregulation. Diabetes Metab.

[b30] Lago F, Gomez R, Gomez-Reino JJ, Dieguez C, Gualillo O (2009). Adipokines as novel modulators of lipid metabolism. Trends Biochem. Sci.

[b31] Moretti C, Frajese GV, Guccione L, Wannenes F, De Martino MU, Fabbri A, Frajese G (2005). Androgens and body composition in the aging male. J. Endocrinol. Invest.

[b32] Pedersen SB, Kristensen K, Hermann PA, Katzenellenbogen JA, Richelsen B (2004). Estrogen controls lipolysis by up-regulating alpha2A-adrenergic receptors directly in human adipose tissue through the estrogen receptor alpha. Implications for the female fat distribution. J. Clin. Endocrinol. Metab.

[b33] Rasouli N, Molavi B, Elbein SC, Kern PA (2007). Ectopic fat accumulation and metabolic syndrome. Diabetes Obes. Metab.

[b34] Rejnmark L (2013). The ageing endocrine system: Fracture risk after initiation of antihypertensive therapy. Nat. Rev. Endocrinol.

[b35] Reusch JE, Klemm DJ (2002). Inhibition of cAMP-response element-binding protein activity decreases protein kinase B/Akt expression in 3T3-L1 adipocytes and induces apoptosis. J. Biol. Chem.

[b36] Sattar N, Wannamethee G, Sarwar N, Chernova J, Lawlor DA, Kelly A, Wallace AM, Danesh J, Whincup PH (2009). Leptin and coronary heart disease: prospective study and systematic review. J. Am. Coll. Cardiol.

[b37] Simoni M, Gromoll J, Nieschlag E (1997). The follicle-stimulating hormone receptor: biochemistry, molecular biology, physiology, and pathophysiology. Endocr. Rev.

[b38] Sun L, Peng Y, Sharrow AC, Iqbal J, Zhang Z, Papachristou DJ, Zaidi S, Zhu LL, Yaroslavskiy BB, Zhou H, Zallone A, Sairam MR, Kumar TR, Bo W, Braun J, Cardoso-Landa L, Schaffler MB, Moonga BS, Blair HC, Zaidi M (2006). FSH directly regulates bone mass. Cell.

[b39] Tajar A, Huhtaniemi IT, O'Neill TW, Finn JD, Pye SR, Lee DM, Bartfai G, Boonen S, Casanueva FF, Forti G, Giwercman A, Han TS, Kula K, Labrie F, Lean ME, Pendleton N, Punab M, Vanderschueren D, Wu FC, Group E (2012). Characteristics of androgen deficiency in late-onset hypogonadism: results from the European Male Aging Study (EMAS). J. Clin. Endocrinol. Metab.

[b40] Trujillo ME, Scherer PE (2006). Adipose tissue-derived factors: impact on health and disease. Endocr. Rev.

[b41] Virtue S, Vidal-Puig A (2010). Adipose tissue expandability, lipotoxicity and the Metabolic Syndrome–an allostatic perspective. Biochim. Biophys. Acta.

[b42] Wang Y, Sullivan S, Trujillo M, Lee MJ, Schneider SH, Brolin RE, Kang YH, Werber Y, Greenberg AS, Fried SK (2003). Perilipin expression in human adipose tissues: effects of severe obesity, gender, and depot. Obes. Res.

[b43] Yang WS, Lee WJ, Funahashi T, Tanaka S, Matsuzawa Y, Chao CL, Chen CL, Tai TY, Chuang LM (2001). Weight reduction increases plasma levels of an adipose-derived anti-inflammatory protein, adiponectin. J. Clin. Endocrinol. Metab.

[b44] Zamboni M, Mazzali G, Zoico E, Harris TB, Meigs JB, Di Francesco V, Fantin F, Bissoli L, Bosello O (2005). Health consequences of obesity in the elderly: a review of four unresolved questions. Int. J. Obes.

[b45] Zuk PA, Zhu M, Ashjian P, De Ugarte DA, Huang JI, Mizuno H, Alfonso ZC, Fraser JK, Benhaim P, Hedrick MH (2002). Human adipose tissue is a source of multipotent stem cells. Mol. Biol. Cell.

