# Supplementary figures and images for: FSH regulates fat accumulation and redistribution in aging through the Gαi/Ca2+/CREB pathway
Source: Aging Cell. 2015 Mar 6;14(3):409–20. doi: 10.1111/acel.12331 (PMC4406670; doi:10.1111/acel.12331)

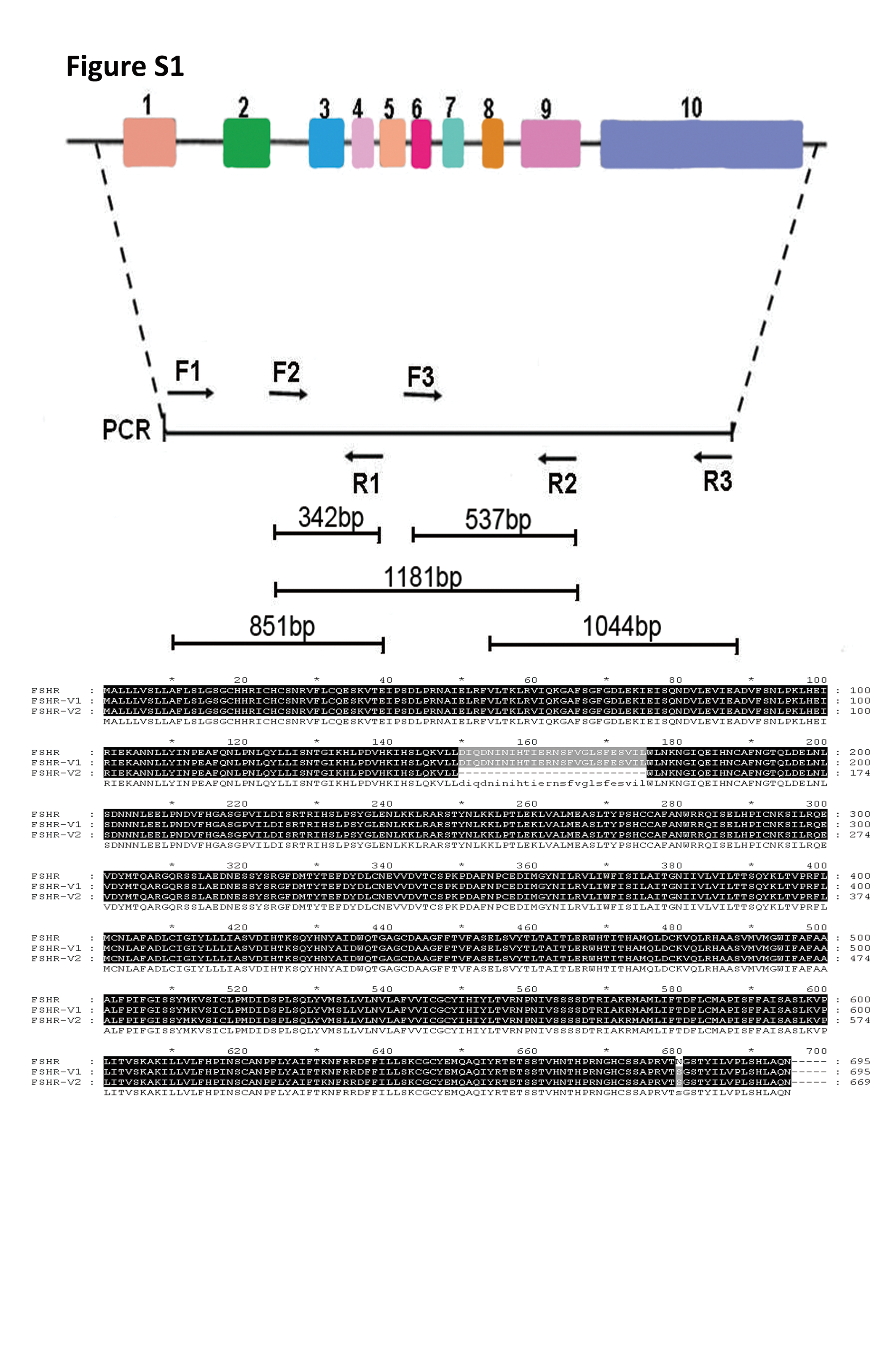

Supplement: Supplementary file 1 [file acel0014-0409-sd1.tif]

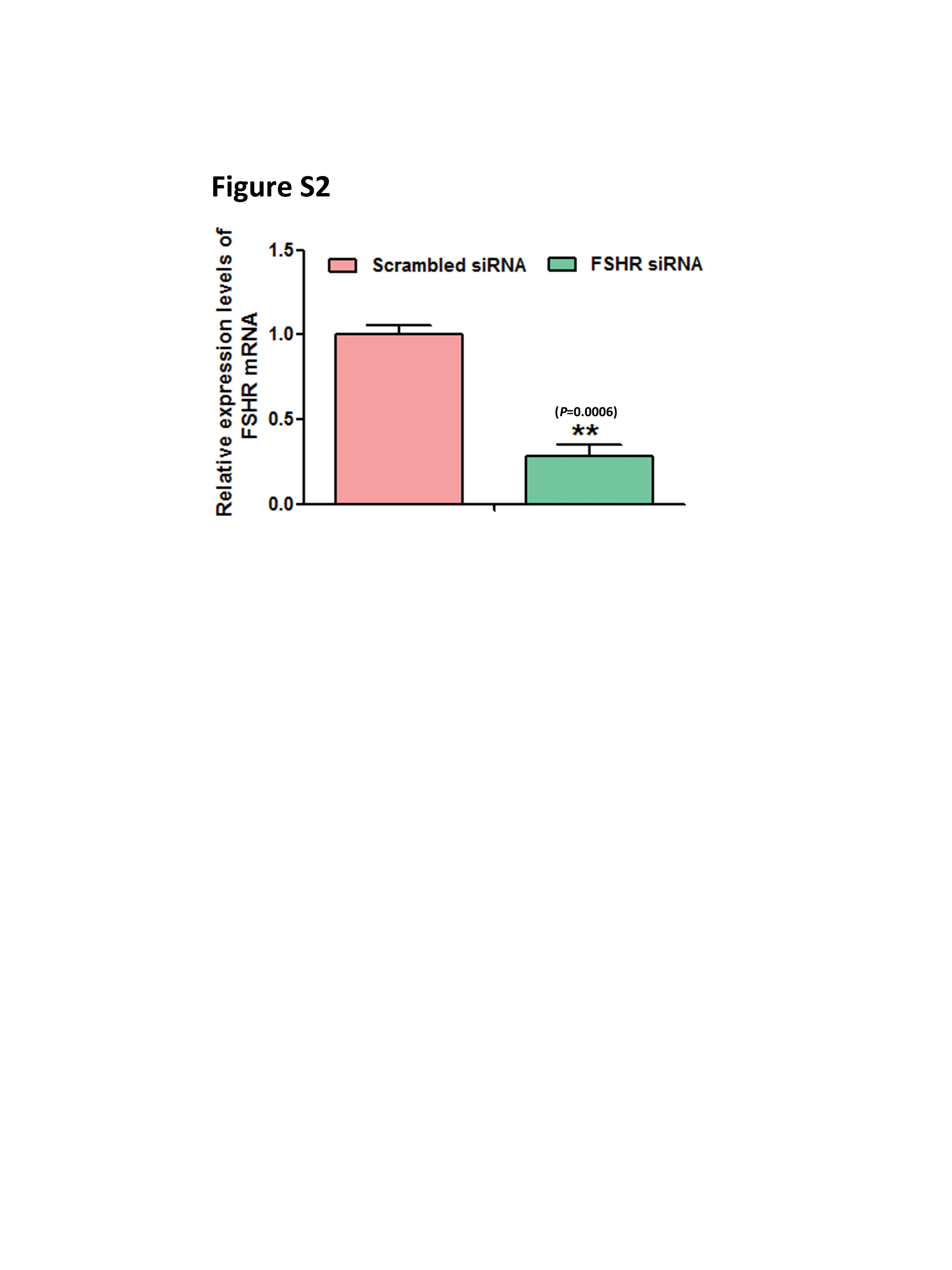

Supplement: Supplementary file 2 [file acel0014-0409-sd2.tif]

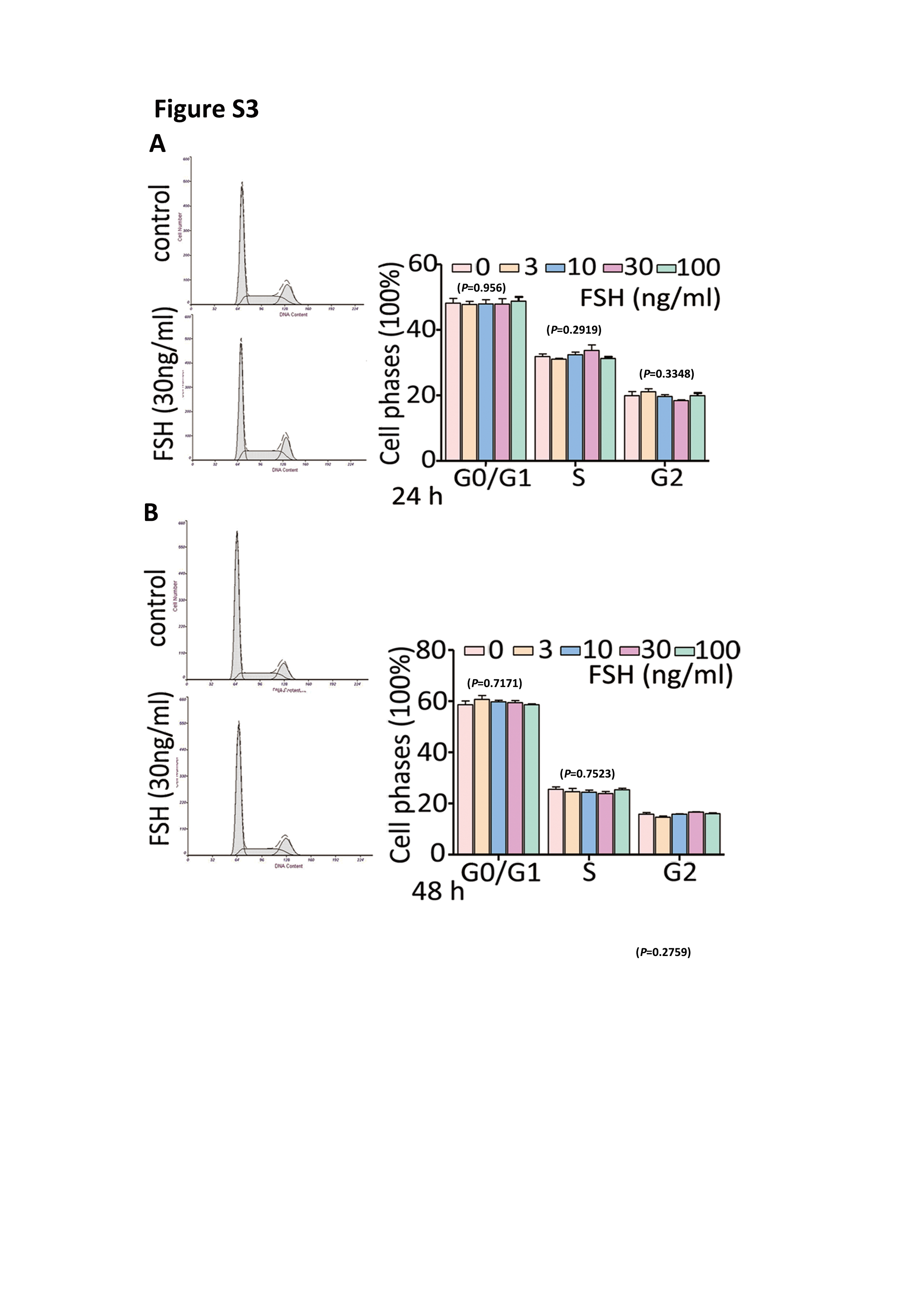

Supplement: Supplementary file 3 [file acel0014-0409-sd3.tif]

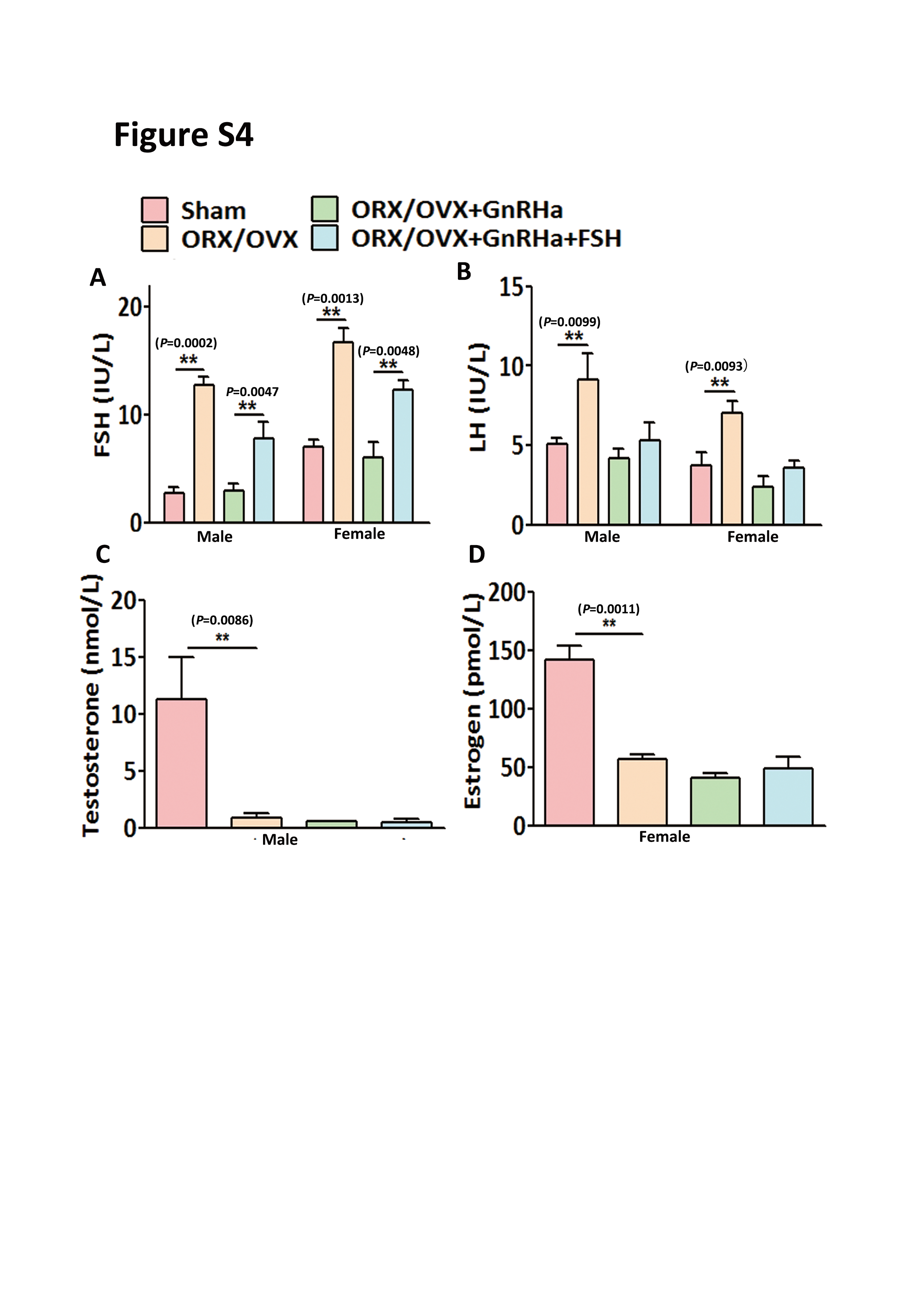

Supplement: Supplementary file 4 [file acel0014-0409-sd4.tif]

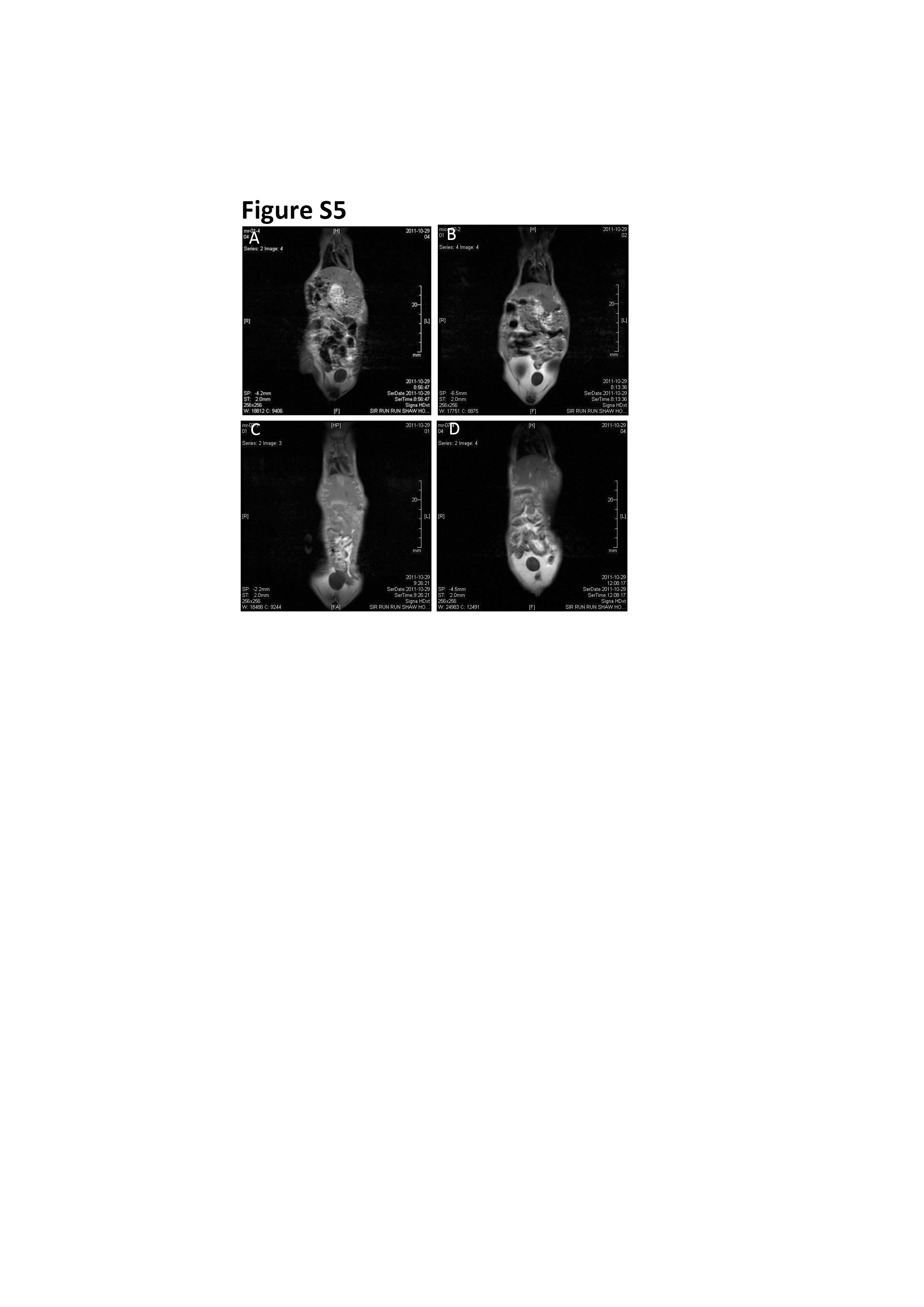

Supplement: Supplementary file 5 [file acel0014-0409-sd5.tif]

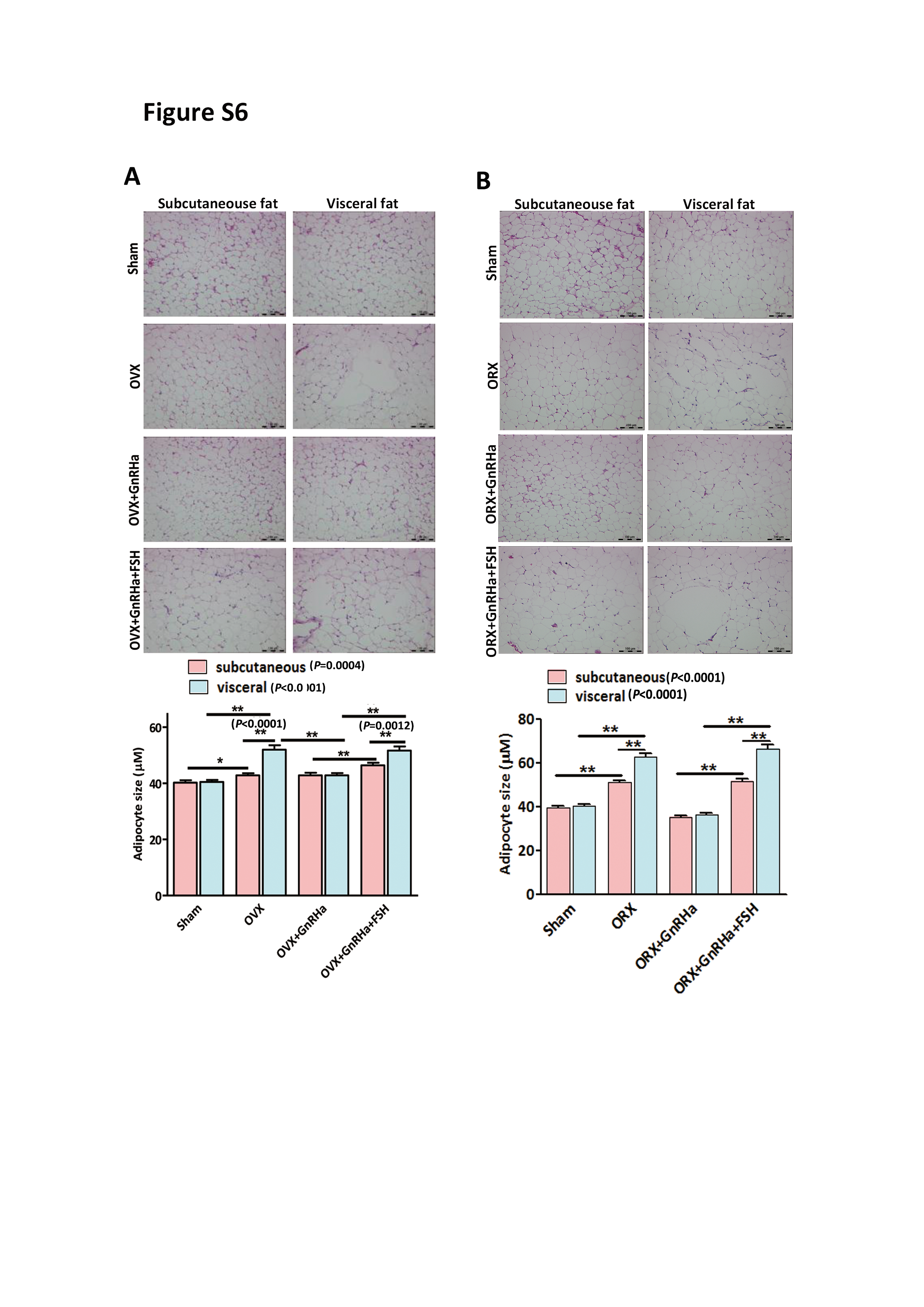

Supplement: Supplementary file 6 [file acel0014-0409-sd6.tif]

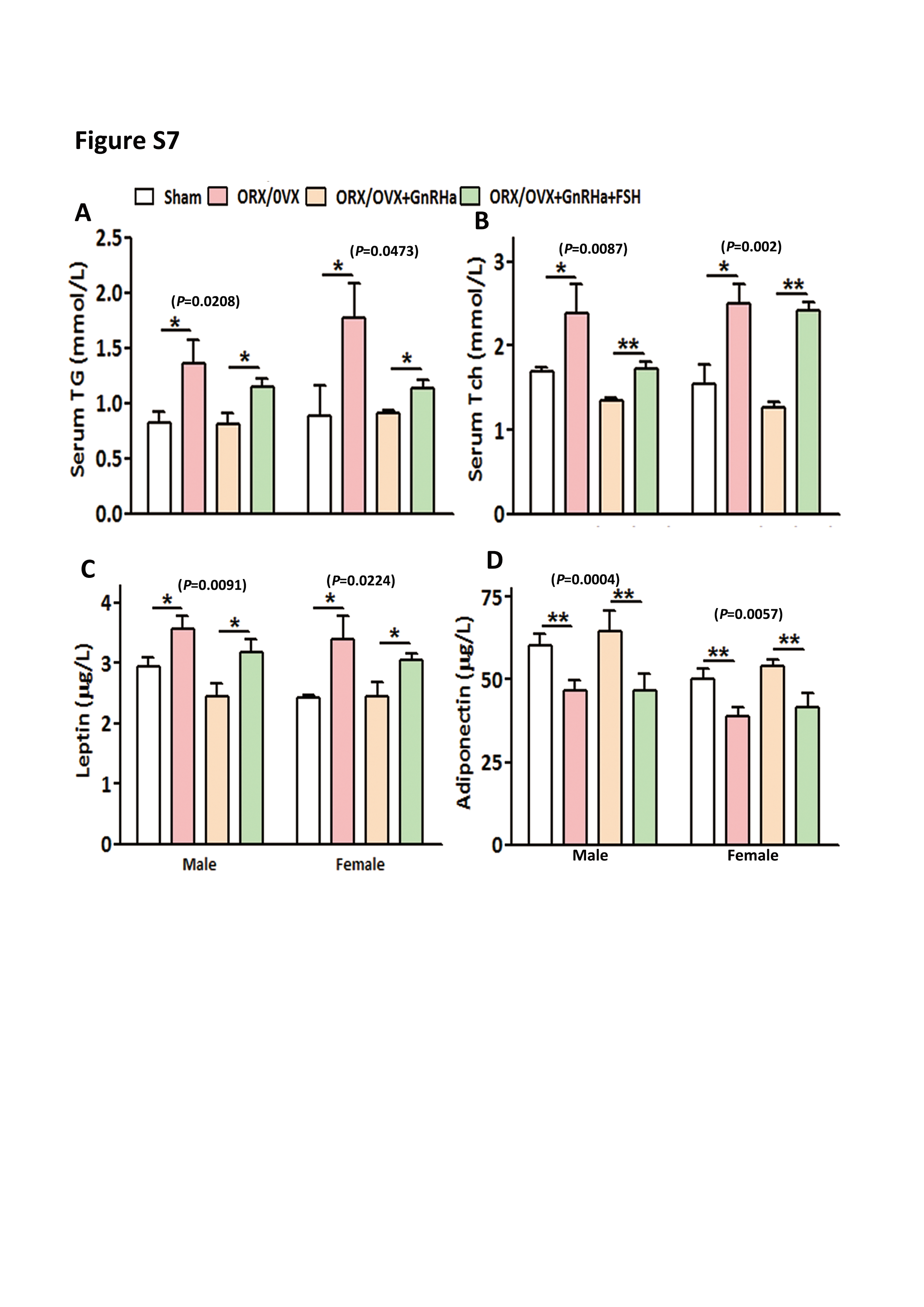

Supplement: Supplementary file 7 [file acel0014-0409-sd7.tif]

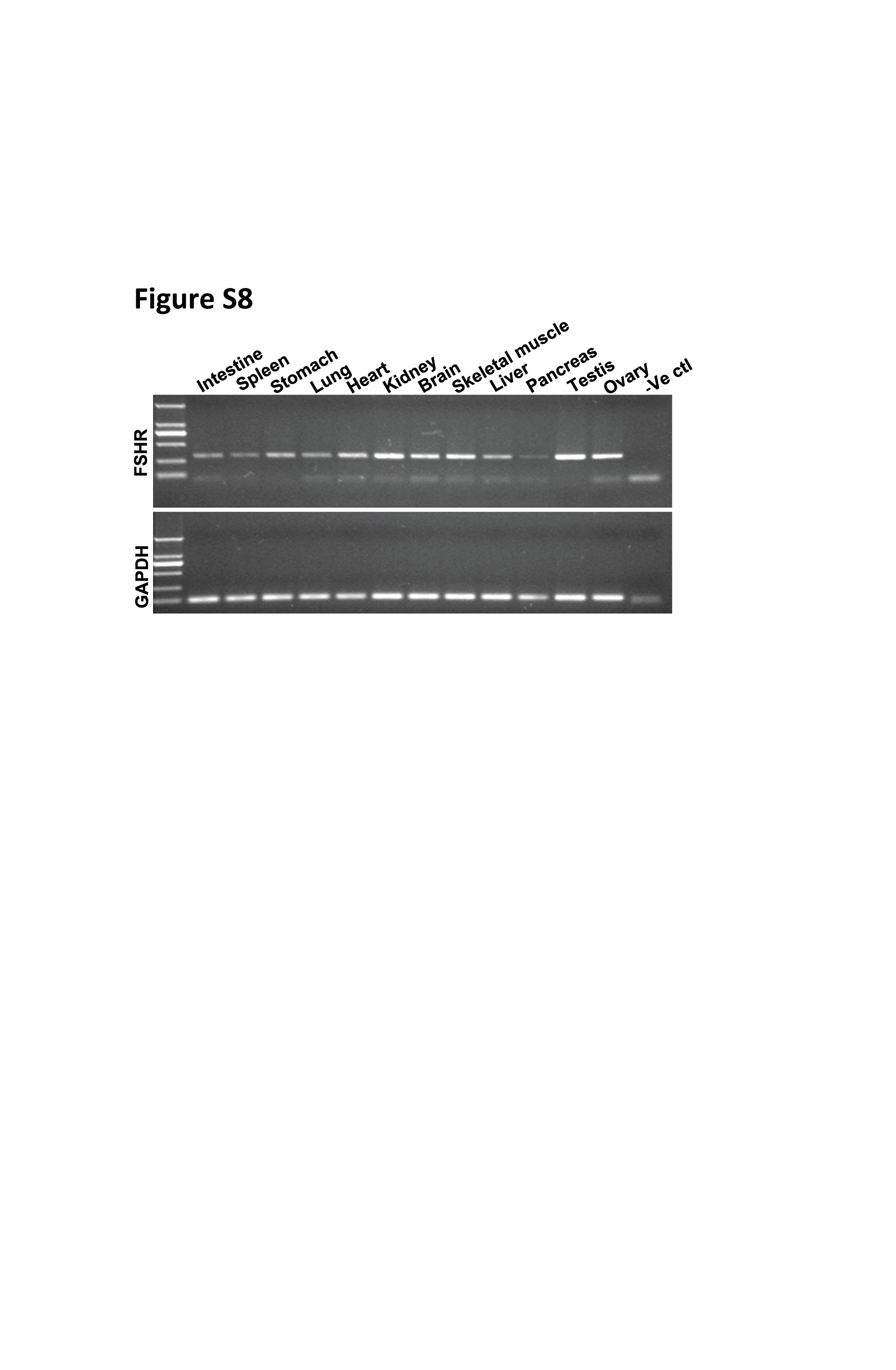

Supplement: Supplementary file 8 [file acel0014-0409-sd8.tif]
