# Supplementary material for: FSH regulates fat accumulation and redistribution in aging through the Gαi/Ca2+/CREB pathway
Source: Aging Cell. 2015 Mar 6;14(3):409–20. doi: 10.1111/acel.12331 (PMC4406670; doi:10.1111/acel.12331)
Supplement: Supplementary file 9 [file acel0014-0409-sd9.doc]

**Supporting information**

1. **Supporting experimental procedures**

**Cloning of FSH receptor full-length cDNA in human adipocytes**

Total RNA was isolated using RNAiso Reagent (TaKaRa, Japan) and the complete coding region of human FSHR cDNA was synthesized using oligo-dT and random primers (TaKaRa, Japan). Using oligo primers (described in Table S3), the PCR reaction included a first denaturing cycle at 95°C for 5 min, followed by 30 cycles of amplification defined by denaturing at 95 °C for 1 min, annealing at 55°C for 1 min, and extension at 72°C for 1.5 min, final extension cycle of 72°C for 10 min. Five different size of DNA fragments were obtained and purified by Agarose Gel DNA purification kit (TaKaRa, Japan) and finally eluted in 15μl ddH2O. The purified PCR products were cloned and sequenced using the pMD19-T Vector System (TaKaRa, Japan). The sequence obtained by cloning/sequencing of PCR products was analyzed by using 3730 DNA Analyzer Polymers (Applied Biosystems). Analysis of nucleotide sequence and predicted amino acid composition was performed using the DNAman program (Lynnon Biosoft). To avoid PCR-derived sequence errors, positive alignment of at least three independent sequences was required.

**Quantitative reverse transcription-polymerase chain reaction**

Total RNA was extracted by Trizol reagent (TakaRa, Japan) and reverse-transcribed to cDNA as described in the manufactures guidelines. We carried out PCR with specific primers (Table S4) on ABI Prism 7900HT (Applied Biosystems, Foster City, CA). The amplification thermal cycling conditions were: 95 °C for 10 sec for one cycle, 95 °C for 5 sec and 60 °C for 30 sec, followed by 40 cycles. We detemined relative mRNA expression of pro-adipogenic genes, including PPARγ (proliferator-activated receptor gamma), C/EBPα (CAAT/enhance binding proteins α), FAS (fatty acid synthase), LPl (Lipoprotein lipase) and perilipin, by the 2−ΔΔCT method normalized to GAPDH levels.

**Small interfering RNA (siRNA)**

For FSHR RNAi studies, cultured human pre-adipocytes and 3T3-L1 pre-adipocytes were transiently transfected with 7nM small interfering RNA (siRNA ID: s66239, s66241, Ambion, Austin, TX) to target FSHR, or, FAM-labeled, scramble control siRNA (Ambion, Austin, TX) using Lipofectamine 2000 reagent according to the manufactures guidelines (Invitrogen, Carlsbad, CA) for 24 h, before subjecting them to FSH treatment.

**Western blot analysis**

We homogenized adipose tissue samples in 1× RIPA buffer containing 0.1 mmol/L PBS, 1% Nonidet P-40, 0.1% SDS, 0.05% deoxycholate, and protease inhibitors (100 μg/mL Leupeptine and 100μg/mL PMSF). The homogenate was incubated on ice for 30 min and centrifuged at 12,000g for 15 min. We determined the protein concentration in the supernatant by Bradford Assay (Bio-Rad Laboratories, Hercules, CA). 50 μg of protein were added to 6× SDS buffer and denatured at 95 °C for 5 min. Samples (50μg/lane) were separated on a 10% SDS-polyacrylamide gel and transferred to nitrocellulose membrane (Immobilon; Millipore, Bedford, MA). After blocking in 5% milk in TBS-T (0.05% Tween 20 in TBS) for 1 h, we blotted the membranes with rabbit anti-FSHR antibody (1:500), rabbit anti-CREB (1:500, Cell signaling technology, Danvers, MA), rabbit anti-phospho-CREB (1:200, Cell signaling technology, Danvers, MA), rabbit anti-PPARγ (1:500, Cell signaling technology, Danvers, MA), rabbit anti-Actin antibody (1:5000, Santa Cruz Biotechnology, CA) or rabbit anti-β-tubulin (1:2000, Santa Cruz Biotechnology, CA) at 4°C overnight. After several washes with TBS-T, the membranes were incubated with appropriate secondary antibody for 1 h at room temperature. We visualized the blots using enhanced chemiluminescence detection reagent (Santa Cruz Biotechnology, Santa Cruz, CA). Human granulosa cells acted as positive controls for FSHR.

**Measurement of TG, Tch, leptin and adiponectin concentrations in human and mice**

Serum triglyceride (TG) and total cholesterol (Tch) levels in mice we detected by chemiluminescence. Concentrations of leptin and ADPN in culture medium of human adipocytes were measured by ELISA using commercially available kits (R&D System, Inc., Minneapolis MN) according to the instructions of the manufacturers. Serum FSH, luteinizing hormone (LH), testosterone (T), and Estrogen (E2) levels were measured by radioimmunoassay. All samples were run in duplicate and assayed at the same time to avoid inter-assay variations or possible changes due to freezing and thawing.

**Measurement of intracellular Ca^2+^**

Cells grown on 25 mm diameter glass coverslips were washed 3 times with Kreb’s solution to remove the culture medium. They were then incubated in Kreb’s solution containing 3 M Fura-2 and 1.6 M Pluornic F-127 for 45 min at 37°C. We then washed the cells twice and kept them in Kreb’s solution for 20 min at 37°C. Coverslips were transferred on a mini-chamber and placed on the stage of an epifluorescence microscope (Nikon Eclipse Ti, Japan) equipped with a CCD camera (Spot Xplorer, USA) and a Fluor 20 objective lens (0.75 NA) (Nikon, Japan). A dual excitation at 340 and 380 nm was used and emissions collected at 510 nm before imaging with MetaFluor (Universal Imaging). Intracellular Ca2+ changes are presented as the change in F340/F380 ratio.

1. **Supporting Figure legends**

**Figure S1- Molecular cloning of human FSHR gene coding sequence (CDS) in human adipocytes.**

The deduced amino-acid sequence alignment of human adipocyte FSHR with the human FSHR isoform. The nucleotide sequences have been submitted to the GenBank, and, are available under the accession number JN003607.

**Figure S2- Relative expression of FSHR mRNA in 3T3-L1 pre-adipocytes treated by specific FSHR siRNA (7nM).**

**Figure S3- Effects of FSH on 3T3-L1 cell cycle progression.**

**(A)** Cell cycle progression in 3T3-L1 cells treated with FSH at different concentrationsfor 24 h or **(B)** 48 h. All values are mean ± s.e.m. No significant differences among groups.

**Figure S4- Serum levels of FSH, LH, testosterone and estogen after gonadectomy.**

(A) Serum FSH, (B) LH, (C) testosterone and (D) estrogen levels in male and female mice after ovariectomy, orchiectomy plus GnRHa administration with or without FSH. All values are mean±s.e.m. (n = 5 for a-d). * *P*<0.05 and ** *P*<0.01.

**Figure S5- Coronal T1-weighted spin-echo MR images obtained with volume segmentation for intra-abdominal for total adipose tissue.** (A, sham group; B, ORX group; C, ORX+GnRHa group; and d, ORX+GnRHa+FSH group)

**Figure S6- H-E staining of subcutaneous and visceral adipose tissue in different treatment groups with corresponding measurement of cell size in male mice.** A for female and B for male**,** all values are mean ± s.e.m. n = 5. * *P*<0.05 and ** *P*<0.01.

**Figure S7- Lipid metabolism of mice after gonadectomy.**

**(A)** Serum triglyceride (TG), **(B)** total cholesterol (Tch), **(C)**leptin and **(D)** adiponectin (ADPN) levels in male and female mice after gonadectomy, or gonadectomy plus GnRHa administration with or without FSH. All values are mean ± s.e.m. (n = 5 for **A-D**). **P*<0.05 and ***P*<0.01.

**Figure S8- Expression of FSHR mRNA in difference tissues.**

1. **Supporting Tables**

Table S1 primers in molecular cloning of human FSHR coding sequence

| **Forward primer** | **Reverse primer** |
| --- | --- |
| CAAATGCAGGAAGAAATCAG (F1) | ATTCCTTGGATGGGTGTTGTG (R1) |
| GTGCGGAACCCCAACATCGTG (F2) | CAAAGGGCTGTCAATATCCA (R2) |
| CCTAAGCCAGATGCATTCAA (F3) | GTTTTGGGCTAAATGACTTA (R3) |
