# Supplementary material for: FSH regulates fat accumulation and redistribution in aging through the Gαi/Ca2+/CREB pathway
Source: Aging Cell. 2015 Mar 6;14(3):409–20. doi: 10.1111/acel.12331 (PMC4406670; doi:10.1111/acel.12331)
Supplement: Supplementary file 10 [file acel0014-0409-sd10.doc]

**Supplementary Figures Legends**

**Figure S1- Molecular cloning of human FSHR gene coding sequence (CDS) in human adipocytes.**

The deduced amino-acid sequence alignment of human adipocyte FSHR with the human FSHR isoform. The nucleotide sequences have been submitted to the GenBank, and, are available under the accession number JN003607.

**Figure S2- Relative expression of FSHR mRNA in 3T3-L1 pre-adipocytes treated by specific FSHR siRNA (7nM).**

**Figure S3- Effects of FSH on 3T3-L1 cell cycle progression.**

**(A)** Cell cycle progression in 3T3-L1 cells treated with FSH at different concentrationsfor 24 h or **(B)** 48 h. All values are mean ± s.e.m. No significant differences among groups (N=5).

**Figure S4- Serum levels of FSH, LH, testosterone and estogen after gonadectomy.**

(A) Serum FSH, (B) LH, (C) testosterone and (D) estrogen levels in male and female mice after ovariectomy, orchiectomy plus GnRHa administration with or without FSH. All values are mean±s.e.m. (n = 5 for a-d). * *P*<0.05 and ** *P*<0.01.

**Figure S5- Coronal T1-weighted spin-echo MR images obtained with volume segmentation for intra-abdominal for total adipose tissue.** (A, sham group; B, ORX group; C, ORX+GnRHa group; and d, ORX+GnRHa+FSH group)

**Figure S6- H-E staining of subcutaneous and visceral adipose tissue in different treatment groups with corresponding measurement of cell size in female (A) and male (B) mice.** All values are mean ± s.e.m. n = 5. * *P*<0.05 and ** *P*<0.01.

**Figure S7- Lipid metabolism of mice after gonadectomy.**

**(A)** Serum triglyceride (TG), **(B)** total cholesterol (Tch), **(C)**leptin and **(D)** adiponectin (ADPN) levels in male and female mice after gonadectomy, or gonadectomy plus GnRHa administration with or without FSH. All values are mean ± s.e.m. (n = 5 for **A-D**). **P*<0.05 and ***P*<0.01.

**Figure S8- Expression of FSHR mRNA in difference tissues.**
